# ZC3H12D and DDX5 Antagonistically Regulate Cyclin D1 mRNA Stability and Cell Cycle Progression in Breast Cancer

**DOI:** 10.1002/cam4.71396

**Published:** 2025-11-20

**Authors:** Liang Sun, Xueting Liu, Wenbao Lu

**Affiliations:** ^1^ Department of Laboratory Medicine The Affiliated Guangdong Second Provincial General Hospital of Jinan University Guangzhou Guangdong China; ^2^ Institute of Microcirculation Chinese Academy of Medical Sciences & Peking Union Medical College Beijing China

**Keywords:** breast cancer, cell cycle, cyclin D1, DDX5, ZC3H12D

## Abstract

**Background:**

An imbalance between the expression of cell cycle‐promoting and cell cycle‐inhibiting genes triggers uncontrolled cell cycle progression in cancer cells. However, the mechanism controlling the expression of cell cycle‐related genes, especially those whose expression is mediated by RNA‐binding proteins (RBPs), remains elusive.

**Methods:**

All RBP expression in human breast cancer was analyzed by bioinformatic methods. The expression and prognostic value of RBPs ZC3H12D (zinc finger CCCH domain‐containing protein 12D) and DDX5 (DEAD box protein 5) in breast cancer were analyzed in public databases and tumor samples. The functions of ZC3H12D and DDX5 in breast tumor cell cycle regulation and tumor progression were determined in vitro and in vivo. RNA sequencing, infrared crosslinking immunoprecipitation and RNA sequencing (irCLIP‐Seq), RNA immunoprecipitation (RIP), luciferase assay, mRNA stability detection, protein pull‐down, RNA pull‐down, mass spectrometry, immunocytochemistry, RNA‐EMSA, and RNA immunoprecipitation chromatin immunoprecipitation (RIP‐ChIP) were conducted to determine the underlying molecular mechanisms of ZC3H12D and DDX5.

**Results:**

The expression of ZC3H12D and DDX5 was reduced and increased in human breast cancer tissues, respectively, and was closely related to the prognosis of breast cancer patients. ZC3H12D and DDX5 could inhibit or promote breast tumor progression by reciprocally controlling the G1/S transition of tumor cells. Mechanistically, ZC3H12D destabilized the CCND1 mRNA by directly binding the conserved stem–loop structure localized in the 3′‐untranslated region (3′UTR) via the RNase domain. Ribosomal protein L4 (RPL4) was critical for ZC3H12D‐mediated mRNA degradation. Besides, DDX5 was shown to bind the common RNA stem–loop structure of CCND1 but increased its stability.

**Conclusions:**

ZC3H12D and DDX5 are a pair of cell cycle antagonists in breast cancer that inhibit or promote the cell cycle progression by modulating the expression of cell cycle‐promoting genes, which provide new insights into the prevention of uncontrolled cancer cell cycle transitions.

## Graphical abstract

Here, our findings demonstrate that RNA‐binding proteins (RBPs) ZC3H12D and DDX5 antagonize breast tumor cell cycle progression by competitively regulating the mRNA stability and expression of cell cycle‐promoting genes via targeting the RNA stem–loop structure, which broadens our understanding of the RBPs‐mediated post‐transcriptional regulation of cell cycle‐related genes. These findings reveal a novel reciprocal modulation of tumor cell cycle transition and highlight the potential of ZC3H12D and DDX5 as a pair of promising therapeutic targets for breast cancer.

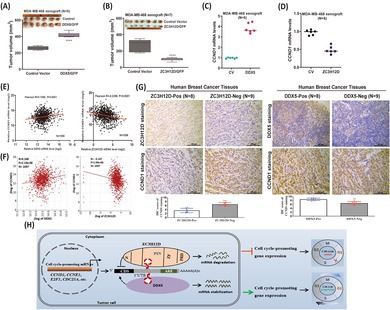

## 1 Background

Breast cancer is a malignant tumor in females with the highest incidence rate in the world. The occurrence of breast cancer is due mainly to the uncontrolled proliferation of breast epithelial cells through the actions of various carcinogenic factors. At present, the complex molecular mechanism of the unlimited proliferation of breast cancer cells is still unclear. Dysregulation of cell cycle progression is one of the reasons for the unlimited proliferation of breast cancer cells [1]. An imbalance in the expression of cell cycle‐promoting and cell cycle‐inhibiting genes within tumor cells is the main cause of uncontrolled cell cycle [2]. Although advances have recently been made in regulating the cell cycle [3], the roles and mechanisms by which RNA‐binding proteins (RBPs) antagonistically regulate cell cycle‐related gene expression are still not completely clear [4].

RBPs can control gene expression by regulating the stability, translation, and alternative splicing of gene transcripts, including cell cycle‐related genes [5]. Zinc finger CCCH domain‐containing protein 12D (ZC3H12D) is an RNA‐binding protein, also known as an endonuclease, capable of binding to and degrading various inflammatory mRNAs [6]. ZC3H12D was previously considered a potential tumor suppressor [7]. The expression of ZC3H12D is absent in lung cancer or follicular lymphoma [8, 9]. In addition, downregulation of ZC3H12D expression promotes the proliferation of osteosarcoma cells [10], indicating the anticancer potential of ZC3H12D in different human cancers. Although the functions of ZC3H12A (a homolog of ZC3H12D) have been extensively studied [11], the roles of ZC3H12D and ZC3H12A are not completely consistent [12, 13]. To date, the role and mechanism of ZC3H12D in human cancer progression, especially in regulating the cell cycle progression of breast tumor cells, are still unknown.

Cyclin D1 (CCND1) is an important cell cycle‐promoting gene that can facilitate the cell cycle transition from G1 phase to S phase (DNA synthesis phase) [14]. The expression of CCND1 is increased in almost all types of human cancers, including breast cancer [15]. CCND1 expression is closely related to the tumorigenesis and progression of breast cancer [16]. However, the mechanism controlling the expression of the CCND1 gene in breast tumor cells is still not fully understood.

In this study, we discovered that the RBPs ZC3H12D and DDX5 (DEAD‐box helicase 5) antagonistically regulate CCND1 mRNA stability and expression in breast tumor cells. Unlike ZC3H12A, which induces the apoptosis of breast tumor cells [17], ZC3H12D mainly induces cell cycle arrest in breast tumor cells by preventing the G1/S transition. ZC3H12D degrades the CCND1 mRNA via the PIN domain by binding the stem–loop structure localized in the 3′ untranslated region (3′UTR). Ribosomal protein L4 (RPL4) was shown to interact with ZC3H12D and facilitate the degradation of the CCND1 mRNA in breast tumor cells. Unexpectedly, we found that the RNA helicase DDX5 [18], another RBP, could bind the common stem–loop structure of CCND1 but stabilize its mRNA. Notably, DDX5 and ZC3H12D could positively and negatively regulate the expression of CCND1, respectively, in human breast tumors. Taken together, these results revealed RBP‐mediated antagonistic regulation of cell cycle‐promoting genes in breast tumor cells, which provided a potential strategy for the future targeted treatment of breast cancer.

## 2 Methods

### 2.1 Cell Culture and Plasmids

Human breast cancer cell lines (MDA‐MB‐468, MDA‐MB‐231, MCF7, and T47D) and human normal mammary epithelial cell lines (MCF‐10A, MCF‐12A) were obtained from the American Type Culture Collection (ATCC; Manassas, VA, USA). HEK293 and HEK293T cells were obtained from the National Infrastructure of Cell Line Resource (Beijing, China). MDA‐MB‐468, MDA‐MB‐231, MCF7, HEK293, and HEK293T cells were cultured in DMEM medium (Gibco, Gaithersburg, MD, USA) with 10% fetal bovine serum (FBS; Gibco, Gaithersburg, MD, USA) plus 1% penicillin/streptomycin (Gibco, Gaithersburg, MD, USA). T47D cells were cultured in PRM1640 medium supplemented with 10% FBS plus 1% penicillin/streptomycin (Gibco, Gaithersburg, MD, USA). MCF‐10A and MCF‐12A were cultured in DMEM medium containing 5% donor horse serum, EGF (20 ng/mL), hydrocortisone (0.5 mg/mL), human recombinant insulin (10 mg/mL), and 1% penicillin/streptomycin (Gibco, Gaithersburg, MD, USA). The human full‐length ZC3H12D coding sequence (NM_207360.2) and DDX5 coding sequence (NM_004396.5) were synthesized, sequenced and inserted into the pEGFP‐N1 vector at NheI/HindIII and XhoI/BamHI sites, respectively. ZC3H12D serial truncations were generated by inserting the PCR‐amplified fragments into the pEGFP‐N1 vector. The luciferase reporters were constructed by inserting the full‐length 3′UTRs of human *CCND1*, *CCNE1*, *CDK2*, and *MCM2* into the pGL3 control vector (Promega, Madison, WI, USA) between Xba I and Fse I sites, respectively. For stem–loop deletion reporters, point mutated *CCND1*‐3′UTRs were amplified, sequenced, and inserted into the pGL3 control vector using the Phusion Site‐Directed Mutagenesis Kit (Thermo Scientific, Waltham, MA, USA).

### 2.2 RNA Sequencing

ZC3H12D/GFP‐overexpressing MDA‐MB‐468 cells, ZC3H12D/GFP‐overexpressing MCF7 cells, DDX5/GFP‐overexpressing MDA‐MB‐468 cells, DDX5/GFP‐overexpressing MCF7 cells, and their GFP‐expressing control cells were used for extracting total RNA. RNA sequencing was performed by Allwegene Technology Inc., Beijing with the PE150 sequencing strategy using an Illumina (SanDiego, California, USA) second‐generation high‐throughput sequencing platform. The reads with inferior quality or adapters were filtered. Clean read data were processed using Tophat2 and Cufflinks software to complete the alignment of transcriptomes. Genes not expressed in any sample were excluded from further analysis. Differentially expressed genes and transcripts were then filtered for false discovery rate (FDR)–adjusted *p* values less than or equal to 0.05. The regulated genes were listed in Tables S1, S5.

### 2.3 Infrared Crosslinking Immunoprecipitation and RNA Sequencing (irCLIP‐Seq)

ZC3H12D/GFP‐overexpressing MDA‐MB‐468 cells and GFP‐expressing control cells were first crosslinked by UV irradiation. The whole cell lysates were pre‐cleared with GFP‐Trap Agarose beads (gta‐20; ChromoTek, Munich, Germany), followed by incubation with GFP‐Trap beads at 4°C for 2 h. The beads were collected by centrifugation and incubated with micrococcal nuclease (N3755; Sigma‐Aldrich, Saint Louis, MO, USA) for 10 min at 37°C. After washing the beads, the beads were then incubated with dye‐labeled 3′‐linker overnight at 16°C. The bound protein–RNA complex was boiled at 70°C for 10 min, and then separated by electrophoresis with 4%–12% Nupage gel (NP0322BOX; Invitrogen, Grand Island, USA). The interest protein–RNA complex was transferred to a nitrocellulose filter membrane and imaged by Odyssey infrared laser imaging system (CLX‐1357; Gene Company Limited, Hong Kong, China). The membrane was cut off and digested with proteinase K (#17916; Thermo Scientific, Waltham, MA, USA). Total RNA was extracted and incubated with 5′‐linker, and then transcribed to cDNA by RT‐PCR. The PCR product was collected and used for library construction. Deep sequencing was completed by Beijing Novogene Bioinformatics Technology Co. Ltd. (Beijing, China). The targeted genes were listed in Table S2.

### 2.4 Cell Cycle Analysis

ZC3H12D‐overexpressing, DDX5‐overexpressing, shZC3H12D or their control cells were collected and washed twice with cold PBS, and then fixed in cold 70% ethanol, respectively. The cells were stained with propidium iodide (20 μg/mL; Sigma‐Aldrich, Saint Louis, MO, USA) and RNase A (0.2 mg/mL; Sigma‐Aldrich, Saint Louis, MO, USA) for 30 min. Then, more than 1 × 10^4^ cells were analyzed by flow cytometry. The results were analyzed with the FlowJo software.

### 2.5 Protein Pull‐Down and Mass Spectrometry

Briefly, whole cell lysates were obtained from ZC3H12D‐GFP fusion protein‐expressing MDA‐MB‐231 cells, and then pre‐cleared with GFP‐Trap Agarose (gta‐20; ChromoTek, Munich, Germany) beads, followed by incubation with GFP‐Trap beads at 4°C for 2 h. Then, the pulled down proteins were resolved by SDS‐PAGE and silver staining. High‐resolution mass spectrometry analysis was performed by Beijing Qinglian Biotech Co. Ltd. (Beijing, China) with RIGOL L‐3000 HPLC System (RIGOL, Beijing, China). The raw data were searched against the homo sapiens database by the Proteome Discoverer 2.4 with Sequest HT (Thermo Scientific, Waltham, MA, USA). The identified proteins were listed in Table S3.

### 2.6 RNA Pull‐Down and Mass Spectrometry

Aliquots of 3′‐biotin‐labeled RNA probes (with stem–loop or without stem–loop) were incubated with streptavidin‐coated Dynabeads (M‐280; Invitrogen, Grand Island, USA) for 2 h at 4°C, then the beads were incubated with total protein extracts of MDA‐MB‐468 cells for another 2 h at 4°C. After washing the beads, the elutes were resolved by SDS‐PAGE followed by silver staining. The unique bands were analyzed by mass spectrometry (LC–MS) performed by Beijing Qinglian Biotech Co. Ltd. (Beijing, China) with RIGOL L‐3000 HPLC System (RIGOL, Beijing, China). The raw data were searched against the homo sapiens database by the Proteome Discoverer 2.4 with Sequest HT. The proteins were listed in Table S4.

### 2.7 mRNA Stability

ZC3H12D‐overexpressing or DDX5‐overexpressing MDA‐MB‐468 cells were treated with actinomycin D (ActD, 5 μg/mL, Sigma, Saint Louis, MO, USA) and 5, 6‐dichlorobenzimidazole riboside (DRB, 5 μg/mL, Sigma, Saint Louis, MO, USA) to block de novo RNA synthesis. Then, the total RNA was extracted at different times and reverse transcribed into cDNA. The half‐life of mRNA was determined by comparing the levels of mRNA before adding ActD and DRB. The relative mRNA level was analyzed by real‐time PCR using the CFX96 Real‐time PCR machine (Bio‐Rad, Hercules, California, USA). All primers are provided in Table S6.

### 2.8 RNA‐EMSA (Electrophoretic Mobility Shift Assay)

EMSA was performed as described previously [17]. Briefly, 3′‐end biotin‐labeled RNA probes were incubated with cell lysates containing ZC3H12D‐GFP fusion protein. The RNA–protein complex was separated and detected with HRP‐conjugated streptavidin according to the kit protocol (#20148; Pierce, Rockford, IL, USA). The RNA probe sequences are listed in Table S6.

### 2.9 Animal Study

Six‐week‐old female BALB/c nude mice were from the Institute of Laboratory Animal Science, Chinese Academy of Medical Sciences (CAMS) & Peking Union Medical College (PUMC) and used for animal studies. MDA‐MB‐468 cells (5 × 10^6^/100 μL PBS) stably expressing ZC3H12D‐GFP, DDX5‐GFP, GFP, or shZC3H12D were injected subcutaneously into the fat pad of the mammary gland of nude mice, respectively. Mice were bred in cages with filter tops in a laminar flow hood in pathogen‐free conditions. The whole lung tissues were collected for HE staining. Tumor size was measured by the formula length × width × height (mm^3^). All animal experimental procedures were approved by the Experimental Animal Care and Ethics Committee of the Institute of Microcirculation, CAMS & PUMC.

### 2.10 Immunohistochemistry (IHC) and Hematoxylin & Eosin (H&E) Staining

Breast tumor tissues or surrounding healthy tissues were subjected to IHC to detect the expression of interested proteins using indicated antibodies. Mouse lung tissues were treated for H&E staining. Zeiss Imaging System (Carl Zeiss Microimaging, Göttingen, Germany) was used to visualize the staining sections.

### 2.11 Statistical Analysis

Data in bar graphs represent mean ± SD of at least three biological repeats. Statistical analysis was performed using Student's *t*‐test by comparing treatment versus vehicle control or otherwise as indicated. Comparisons between groups were analyzed by Prism 9 and made by *t*‐test. Spearman correlation analysis was used to evaluate the relationship between different gene expression levels. *p* < 0.05 was considered to be statistically significant.

## 3 Results

### 3.1 The RNA‐Binding Protein ZC3H12D Is Downregulated in Human Breast Tumor Tissues and Is Positively Related to Patient Survival

**FIGURE 1:**
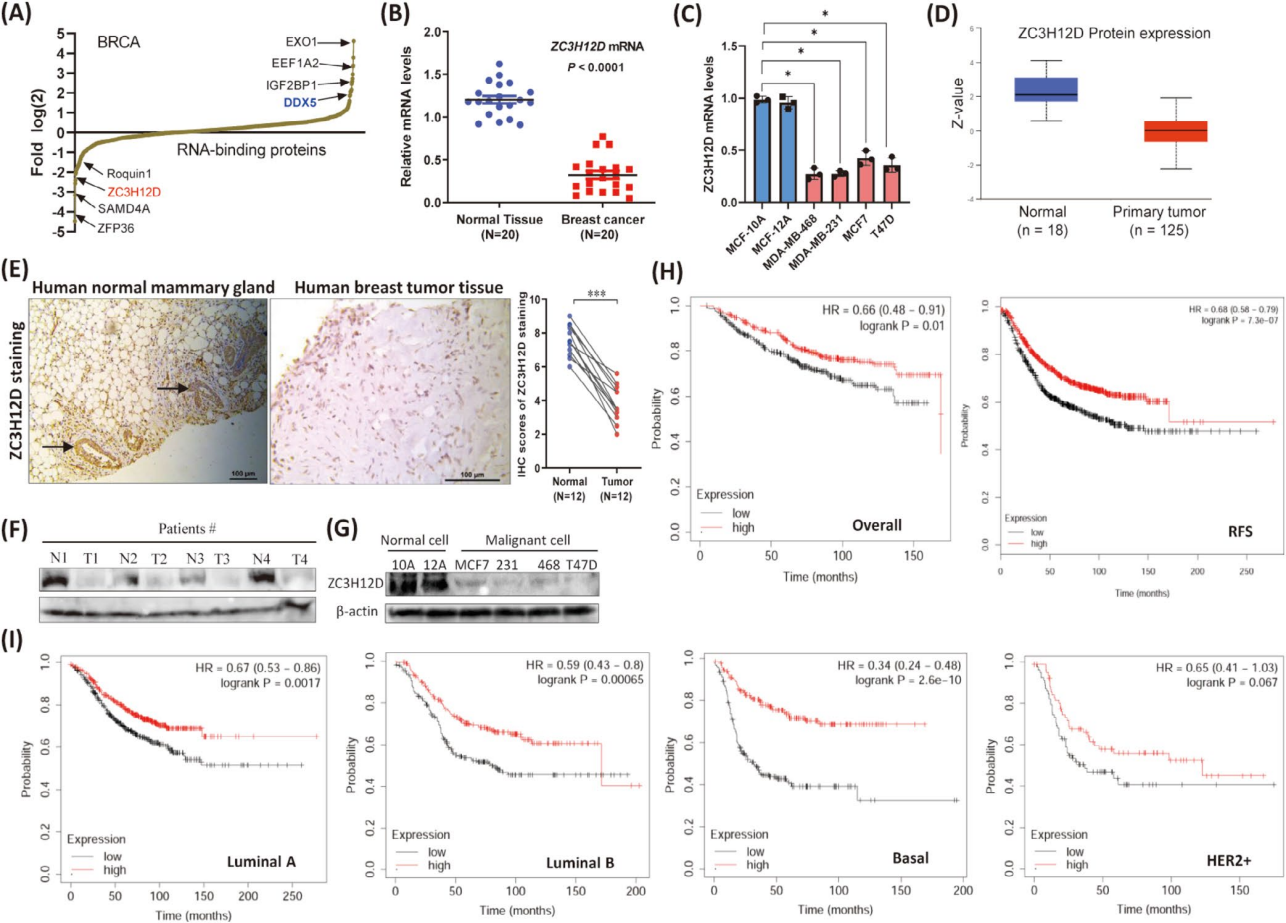
The RNA‐binding protein ZC3H12D is downregulated in human breast tumor tissues and is positively related to patient survival. (A) 1756 RNA‐binding proteins expression in human breast tumors were profiled using the TCGA database. (B) *ZC3H12D* transcript expression in 20 pairs of human breast tumors and normal tissues were assessed by qRT‐PCR. *p* values was determined by student‐*t* test. (C) mRNA expression of levels of ZC3H12D in normal mammary gland cells (MCF‐10A, MCF‐12A) and breast tumor cells (MDA‐MB‐468, MDA‐MB‐231, MCF7, and T47D). (D) ZC3H12D protein expression in human primary breast tumors and normal tissues was displayed based on the UALCAN database (http://ualcan.path.uab.edu/analysis‐prot.html). (E) Representative immunohistochemistry (IHC) images showing ZC3H12D protein expression in normal mammary gland tissues and breast tumor tissues (left). Scale bar, 100 μm. IHC analysis of ZC3H12D staining in 12 pairs of matched breast tumor and normal tissues (right). (F, G) Protein expression levels of ZC3H12D in normal mammary gland tissues and breast tumor tissues (F), and normal mammary gland cells (MCF‐10A, MCF‐12A) and breast tumor cells (MDA‐MB‐468, MDA‐MB‐231, MCF7, and T47D) (G) were measured by western blotting. (H) Overall Survival (OS) and Relapse‐Free Survival (RFS) analyses of breast cancer patients with low and high ZC3H12D expression. (I) RFS curves for ZC3H12D‐low and ZC3H12D‐high luminal A, luminal B, basal, and HER2+ subtypes breast cancer patients. Data are presented as mean ± SD; *p < 0.05, ***p < 0.0001, in unpaired *t*‐test.

We analyzed the expression of 1756 RBPs in breast cancer patients by surveying the TCGA database to systematically investigate the expression of RBPs in human breast cancer [19, 20]. ZC3H12D expression was reduced and ranked among the RBPs with the lowest expression in human breast cancer patients (Figure 1A). Moreover, several RBPs that had been confirmed to be downregulated (such as sterile alpha motif domain‐containing protein 4A (SAMD4A) and Roquin1) [21, 22] or overexpressed (such as insulin‐like growth factor 2 mRNA‐binding protein 1, IGF2BP1) [23] in breast cancer tissues were also identified, which validated our computational analyses. The qRT–PCR results revealed that the ZC3H12D mRNA expression level was significantly reduced in a group of collected breast tumor tissues compared with the adjacent healthy tissues (Figure 1B). *ZC3H12D* expression was also low in several breast cancer cell lines compared with normal breast epithelial cells (Figure 1C). Moreover, ZC3H12D protein levels were also reduced in human breast tumor samples (Figure 1D–F) and cell lines (Figure 1G). We found that different subtypes of breast cancer, including luminal, HER2+, and triple‐negative breast cancer (TNBC), exhibited reduced ZC3H12D expression (Figure S1). Next, we analyzed the correlations between ZC3H12D expression levels and the aggressive characteristics of breast cancer and found that the ZC3H12D protein levels decreased with increasing breast tumor stage (Figure S1). We further showed that breast cancer patients with low ZC3H12D expression experienced shorter Relapse‐free Survival (RFS), Overall Survival (OS), and Distant Metastasis‐Free Survival (DMFS) than did those with high ZC3H12D expression (Figure 1H; Figure S1). Although a statistically significant difference between the expression of ZC3H12D and the survival of HER2+ breast cancer patients was not observed, a similar trend indicated that breast cancer patients with low ZC3H12D expression experienced shorter survival (Figure 1H). Overall, these results indicate that ZC3H12D expression is suppressed in human breast tumors and that its expression is closely related to the progression and prognosis of patients with breast cancer.

### 3.2 ZC3H12D Regulates Cell Cycle Signaling Pathway and Induces G1/S Cell Cycle Arrest in Breast Tumor Cells

**FIGURE 2:**
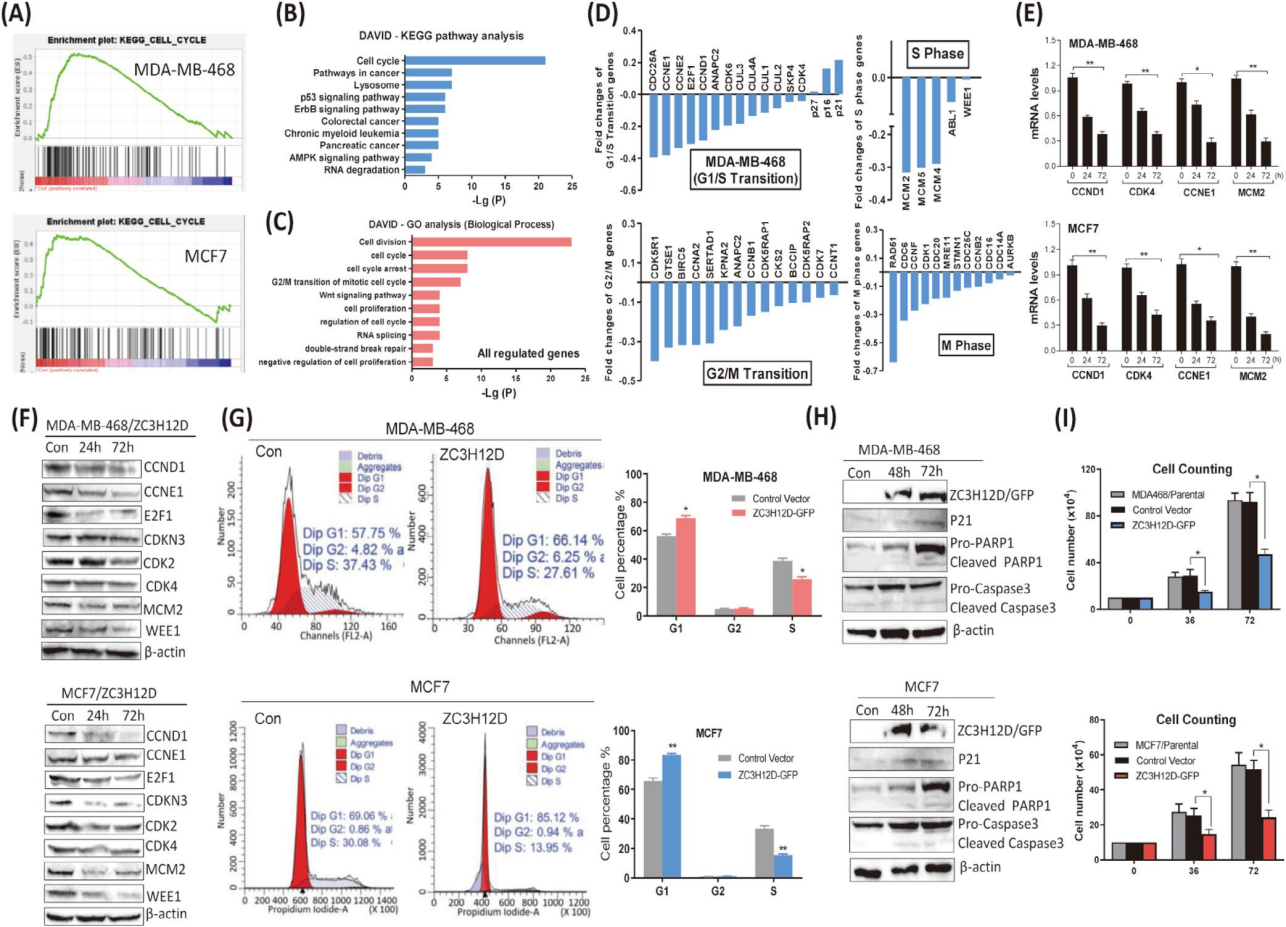
ZC3H12D regulates the cell cycle signaling pathway and induces G1/S cell cycle arrest in breast tumor cells. (A) GSEA enrichment plot indicating that cell cycle pathway is enriched by ZC3H12D in human breast tumor cells. (B) KEGG pathway analysis of differentially expressed genes by ZC3H12D. KEGG pathway analyses were performed using the DAVID tools at (https://david.ncifcrf.gov/). (C) GO (Gene Ontology) analysis of the differentially expressed genes by ZC3H12D was performed using DAVID. (D) Expression of cell cycle‐related genes after overexpression of ZC3H12D in MDA‐MB‐468 cells as measured by RNA‐seq. (E) mRNA expression of indicated cell cycle genes was measured by qRT‐PCR in ZC3H12D‐overexpressing MDA‐MB‐468 and MCF7 cells at different times. (F) Expression of cell cycle‐promoting proteins in ZC3H12D‐overexpressing MDA‐MB‐468 and MCF7 cells as measured by western blotting. (G) FCM analysis showing that cell cycle progression in ZC3H12D‐overexpressing MDA‐MB‐468 and MCF7 cells. Percentages of cells in the G1, G2, and S phase were analyzed. (H) Protein expression of cell cycle inhibitor (p21) and apoptosis indicators (cleaved PARP‐1 and caspase3) were measured by immunoblotting in MDA‐MB‐468 and MCF7 cells after ZC3H12D overexpression. (I) Cell counting showing that cell proliferation of MDA‐MB‐468 and MCF7 cells after ZC3H12D overexpression. Data are shown as mean ± SD; *p < 0.05, **p < 0.001 in unpaired *t*‐test.

To determine the signaling pathway and target genes regulated by ZC3H12D in breast tumor cells, RNA‐sequencing (RNA‐seq) was performed using ZC3H12D‐overexpressing MDA‐MB‐468 and MCF7 cells (Figure S2A). A total of 4319 genes were commonly downregulated, and 3592 genes were commonly upregulated in these two types of breast tumor cells (Figure S2B and Table S1). Kyoto Encyclopedia of Genes and Genomes (KEGG) analysis and Gene Set Enrichment Analyses (GSEA) revealed that the “cell cycle” pathway was the most significantly enriched pathway (Figure 2A,B). Moreover, the Gene Ontology (GO) analysis revealed that the genes regulated by ZC3H12D were significantly enriched in the terms “cell cycle,” “cell division,” and “cell cycle arrest” (Figure 2C). These computational findings indicated that ZC3H12D likely regulates the cell cycle signaling pathway by modulating cell cycle‐related gene expression in human breast tumor cells. Indeed, a series of cell cycle‐promoting genes were specifically downregulated, whereas cell cycle‐inhibiting genes (p27, p21, and p16) were upregulated by ZC3H12D (Figures 2D and S2C). We validated the RNA‐seq results by measuring the mRNA levels of four downregulated cell cycle‐promoting genes, including *CCND1*, *cyclin E1* (*CCNE1*), *cyclin‐dependent kinase 4* (*CDK4*), and *minichromosome maintenance complex component 2* (*MCM2*), via real‐time PCR. The mRNA expression of four cell cycle‐promoting genes was suppressed in breast tumor cells overexpressing ZC3H12D (Figure 2E). Moreover, the protein expression of cell cycle‐promoting genes was also downregulated by ZC3H12D in a time‐dependent manner (Figure 2F). To determine the function of ZC3H12D in breast tumor cell cycle progression, we measured the cell cycle distribution via flow cytometry. As expected, ZC3H12D overexpression significantly increased the percentage of G1 phase cells and reduced the population of S phase cells. A slight increase in the G2 cell population was observed after ZC3H12D overexpression (Figure 2G). Furthermore, ZC3H12D induced the expression of p21, a typical indicator of cell cycle arrest in both types of breast tumor cells (Figure 2H). These findings clearly indicated that ZC3H12D inhibited the G1/S transition of breast tumor cells. In addition, ZC3H12D effectively inhibited the proliferation of breast tumor cells (Figures 2I and S2D). Unlike ZC3H12A, which induces apoptosis in breast tumor cells, ZC3H12D did not substantially increase the levels of cleaved PARP1 and Caspase3, two apoptosis indicators (Figure 2H). We also did not observe a significant increase in the number of floating dead cells among cultured breast tumor cells (Figure S2E). Overall, our results strongly indicate that ZC3H12D regulates the cell cycle signaling pathway and induces G1/S arrest in breast tumor cells.

### 3.3 ZC3H12D Specifically Degrades Cell Cycle‐Promoting mRNAs in a Stem–Loop Structure‐Dependent Manner to Induce Cell Cycle Arrest

**FIGURE 3:**
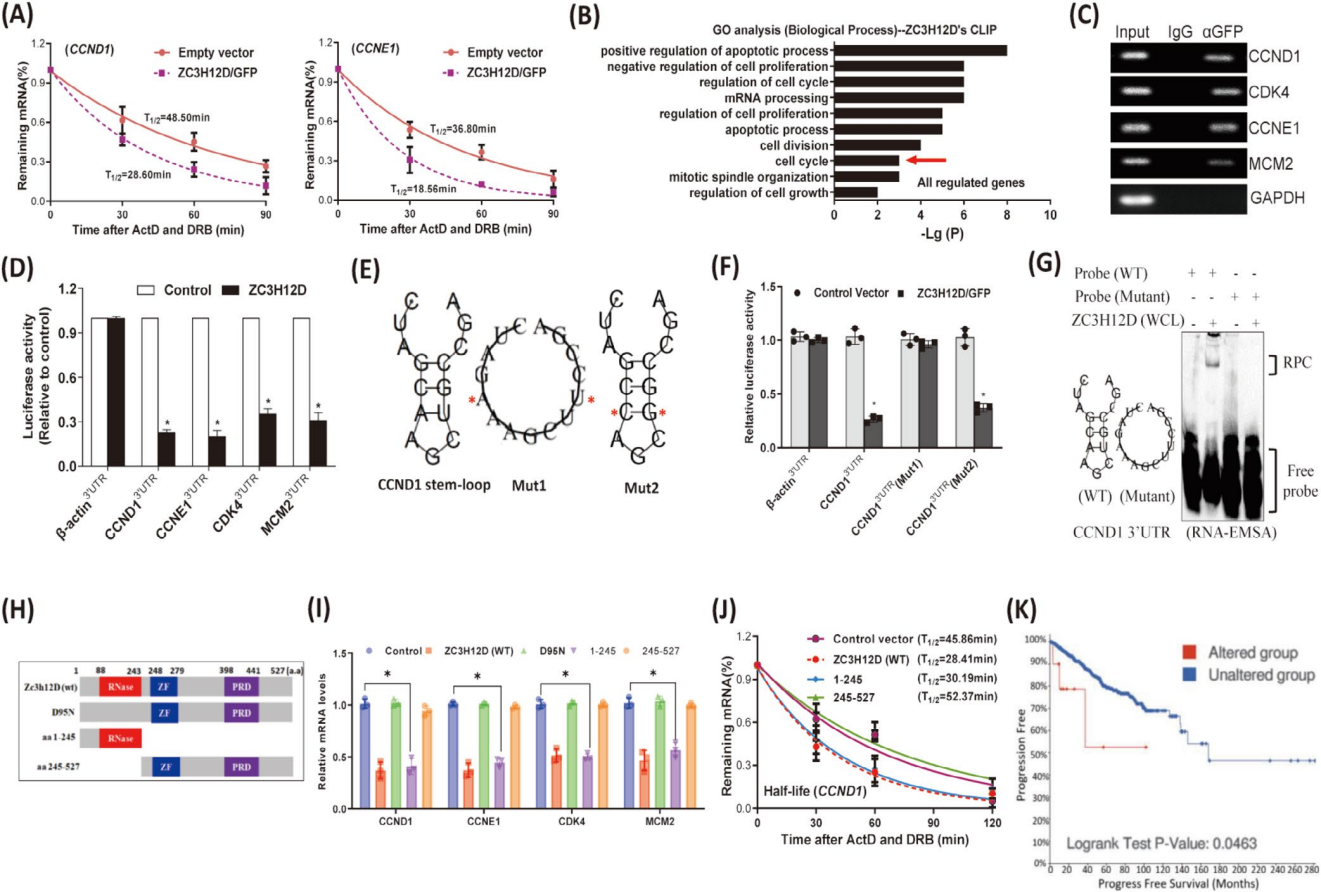
ZC3H12D specifically degrades cell cycle‐promoting mRNAs in a stem–loop structure‐dependent manner to induce cell cycle arrest. (A) Half‐lives of CCND1 and CCNE1 mRNAs were measured in ZC3H12D‐overexpressing MDA‐MB‐468 cells. (B) GO analysis of the bound genes by ZC3H12D was performed using DAVID. (C) RNA‐IP followed by RT‐PCR detection showing that cell cycle‐promoting mRNAs were enriched by ZC3H12D/GFP fusion protein. ZC3H12D/GFP fusion protein were first overexpressed in MDA‐MB‐468 cells, after lysate extraction, immunoprecipitation with anti‐GFP antibody or isotype IgG, and then RNA extraction. (D) Measurement of luciferase activities after transfection with indicated reporters and ZC3H12D expression plasmids in HEX293 cells. β‐actin 3′UTR was used as a negative control. (E) The predicted stem–loop structure in *CCND1* 3′UTR and mutation strategy (red asterisks indicate base substitution). Mut1 cannot be able to form stem–loop structure and Mut2 still maintains stem–loop structure. (F) Measurement of luciferase activities of reporters containing different *CCND1* 3′UTRs. (G) RNA‐EMSA analysis. (H) ZC3H12D wild‐type (WT) and mutants schematics. (I) mRNA expression of indicated cell cycle‐promoting mRNAs was measured by qRT‐PCR in MDA‐MB‐468 cells overexpressing ZC3H12D or its mutants. (J) Half‐lives of CCND1 mRNA were measured after overexpression of ZC3H12D or its mutants in MDA‐MB‐468 cells. (K) Potential correlation between mutation status and progress free survival of breast cancer using the cBioPortal tool. Data are presented as mean ± SD; *p < 0.05 in unpaired *t*‐test.

Previous studies have confirmed that ZC3H12 family proteins function as endonucleases to degrade mRNAs [24]. Therefore, we wondered whether ZC3H12D downregulated the expression of cell cycle‐promoting genes by degrading their mRNAs. Indeed, the half‐lives of cell cycle‐promoting gene mRNAs were shortened in ZC3H12D‐expressing breast tumor cells (Figures 3A and S3A). In contrast, nearly no changes in the half‐lives of cell cycle‐inhibiting mRNAs were observed (Figure S3B). Next, we performed infrared‐CLIP (irCLIP)‐seq to determine whether ZC3H12D directly binds to cell cycle‐promoting mRNAs (Figure S3C) [25]. Interestingly, the GO analysis revealed that the genes bound by ZC3H12D were strongly enriched in the term ‘cell cycle’ (Figure 3B and Table S3), further suggesting a potential association between ZC3H12D and cell cycle‐related mRNAs. We further confirmed that ZC3H12D was able to bind to the cell cycle‐promoting transcripts (*CCND1*, *CDK4*, *CCNE1*, and *MCM2*) by performing RNA immunoprecipitation (RIP) using an anti‐GFP antibody and an isotype IgG followed by RT–PCR detection (Figure 3C). Next, we performed luciferase assays using reporters containing the 3′UTRs of *CCND1*, *CCNE1*, *CDK4*, or *MCM2* to assess whether ZC3H12D regulates cell cycle‐promoting mRNA stability through the 3′UTR (Figure S3D). We showed that ZC3H12D markedly reduced the luciferase activities of all reporters (Figure 3D), indicating ZC3H12D targets the 3′UTRs of cell cycle‐promoting genes.

Among the target mRNAs, CCND1 is an important cell cycle promoter. ZC3H12 family proteins have been reported to degrade mRNAs through stem–loop structures [26]. Here, we showed that a CCND1 3′UTR sequence conserved across different species could be folded into a stem–loop structure (Figures 3E and S3E). Interestingly, ZC3H12D lost its inhibitory effect on luciferase activity once the stem–loop structure was disrupted (Figure 3F). In contrast, restoring the stem–loop structure regardless of sequence changes could trigger the suppression of ZC3H12D again (Figure 3F). We also found that a unique protein–RNA complex formed between ZC3H12D and the RNA probe that contained a stem–loop structure, rather than the linearized mutation probe (Figure 3G). These findings suggest that ZC3H12D decays the mRNAs of cell cycle‐promoting genes in a manner dependent on the stem–loop structure in their 3′UTRs.

Furthermore, to decipher the domain in ZC3H12D responsible for the decay of cell cycle‐promoting mRNAs, three mutants were generated: the D95N mutant (deleted the RNase domain by replacing D with N), amino acid (aa) 1–245 (containing the RNase domain), and aa 245–527 (containing the zinc finger domain and proline‐rich domain) (Figure 3H). The expression of the truncations was confirmed by Western blotting (Figure S3F). As a result, the wild‐type (WT) and aa 1–245 mutant proteins effectively reduced the mRNA expression (Figure 3I) and half‐lives (Figure 3J) of cell cycle‐promoting genes. However, the D95N and aa 245–527 mutants had no effect on the expression or stability of the above mRNAs. These results indicate that ZC3H12D degrades cell cycle‐promoting mRNAs via its RNase domain.

The RNase domain is known to be crucial for ZC3H12 family proteins [26]. We then aimed to clarify the effect of RNase domain inactivation on the progression of human breast cancer. The “missense” and “truncating” mutations of ZC3H12D are the main types of genetic alterations in human breast cancer, and most of the genetic mutations occur in the RNase domain. Moreover, we also found that “missense” is the main mutation in the RNase domain (Figure S3G). Notably, compared with patients without ZC3H12D alterations, those with altered ZC3H12D expression had a worse prognosis in terms of progression‐free survival (Figure 3K). These findings indicate that the genetic mutation of ZC3H12D is common in human breast tumors and is closely related to the prognosis of breast cancer patients.

### 3.4 RPL4 Facilitates the ZC3H12D‐Mediated Decay of Cell Cycle‐Promoting mRNAs in Breast Tumor Cells

**FIGURE 4:**
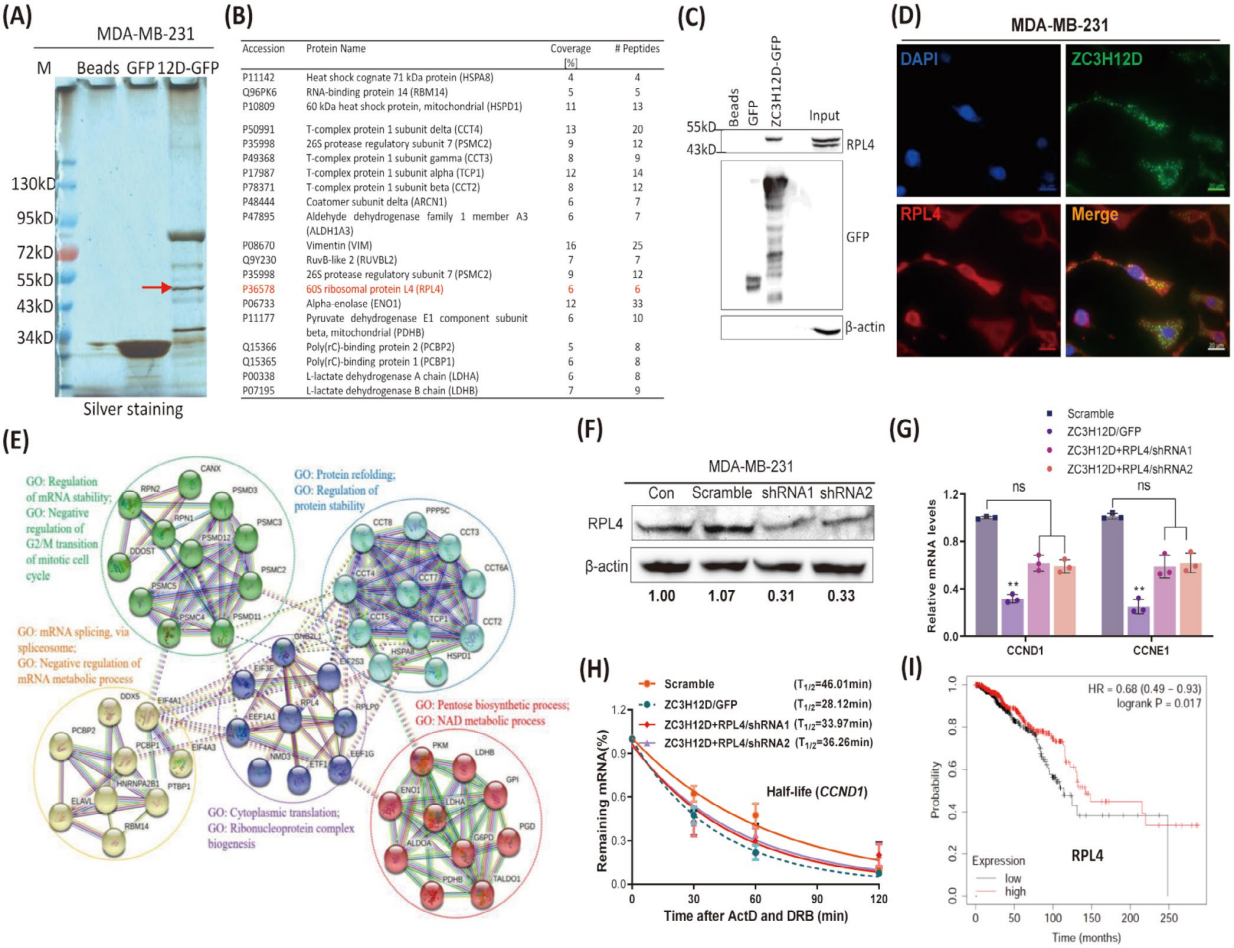
RPL4 facilitates the ZC3H12D‐mediated decay of cell cycle‐promoting mRNAs in breast tumor cells. (A) Silver staining of gel separating ZC3H12D‐interacting proteins. (B) ZC3H12D‐associated proteins identified by mass spectrometry. (C) RPL4 was coprecipitated with ZC3H12D was analyzed by immunoblotting in MDA‐MB‐231 cells. (D) Immunocytochemistry analysis showing the colocalization of ZC3H12D and RPL4 in MDA‐MB‐231 cells. (E) Protein–protein interaction network of ZC3H12D‐associated proteins was analyzed by STRING tool (https://string‐db.org/). (F) Immunoblotting analysis shown that the knockdown of RPL4 in MDA‐MB‐231 cells. (G) CCND1 and CCNE1 mRNAs expression were analyzed by qRT‐PCR after knocking down RPL4 in MDA‐MB‐231/ZC3H12D‐GFP cells. (H) Half‐lives of CCND1 mRNA were measured after knocking down RPL4 in MDA‐MB‐231/ZC3H12D‐GFP cells. (I) Relapse‐free survival curve of breast cancer patients with low and high RPL4 transcripts. Data are presented as mean ± SD; **p < 0.001 in unpaired *t*‐test. ns, Non significance.

Protein–protein interactions affect the ability of RBPs to regulate mRNA stability [27]. To identify the proteins that interact with ZC3H12D in breast tumor cells, protein IP combined with mass spectrometry was performed on MDA‐MB‐231 cells expressing GFP or ZC3H12D‐GFP. Compared with those in the GFP control group, several unique protein bands were observed in the ZC3H12D‐GFP‐overexpressing group (Figure 4A). We screened 20 proteins that are most likely to interact with ZC3H12D based on the molecular weights and number of enriched peptides (Figure 4B and Table S4). Ribosomal proteins were previously shown to interact with ZC3H12A [28], which led us to consider whether ribosomal proteins also interact with ZC3H12D. Therefore, ribosomal protein L4 (RPL4), identified by mass spectrometry, was chosen for further study. Indeed, RPL4 was detected in proteins pulled down from ZC3H12D/GFP‐expressing cells but not from GFP control cells (Figure 4C). The colocalization of ZC3H12D and RPL4 in the cytoplasm was also confirmed by immunohistochemistry (Figure 4D). These results confirmed the interaction between ZC3H12D and RPL4 in breast tumor cells. We speculated that the remaining proteins identified by mass spectrometry might also interact with ZC3H12D; here, we further evaluated the interactions between these proteins using the STRING database (http://string‐db.org). Interestingly, these proteins were significantly enriched into five subgroups. The GO analysis showed that these genes were enriched in terms related to the regulation of mRNA stability, such as “regulation of mRNA stability,” “negative regulation of mRNA metabolic process,” and “ribonucleoprotein complex biogenesis” (Figure 4E). These computational analyses implied that these proteins are likely to affect the mRNA degradation function of ZC3H12D. To verify this hypothesis, RPL4 was knocked down using shRNAs in MDA‐MB‐231 cells overexpressing ZC3H12D and the expression was validated by Western blotting (Figure 4F). Knocking down RPL4 effectively inhibited the ZC3H12D‐induced suppression of the expression (Figure 4G) and stability of the CCND1 mRNA (Figure 4H). Moreover, we also showed that the expression level of *RPL4* was significantly related to the survival of breast cancer patients (Figure 4I). Taken together, these results indicate that RPL4 can interact with ZC3H12D and affect the ability of ZC3H12D to destabilize cell cycle‐promoting mRNAs in human breast tumor cells.

### 3.5 Knocking Down ZC3H12D Increases the Stability and Expression of Cell Cycle‐Promoting mRNAs and Promotes Breast Tumor Growth

**FIGURE 5:**
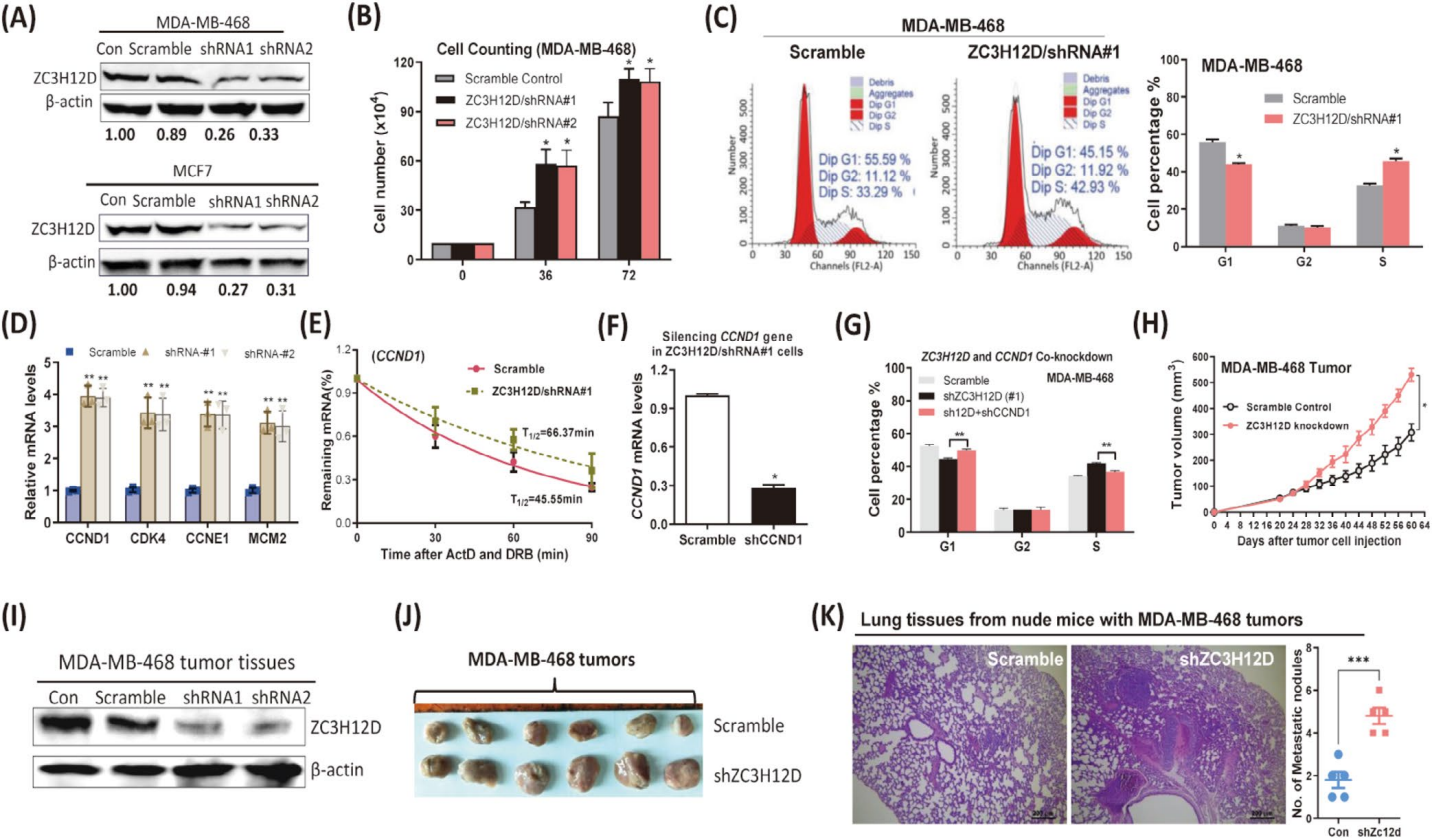
Knocking down ZC3H12D increases the stability and expression of cell cycle‐promoting mRNAs and promotes breast tumor growth. (A) Immunoblotting analysis of ZC3H12D protein expression after infecting ZC3H12D‐shRNAs/lentivirus and scramble/lentivirus in MDA‐MB‐468 and MCF7 cells, respectively. Knockdown quantification was assessed by ImageJ. (B) Cell counting showing that cell proliferation of MDA‐MB‐468 cells after ZC3H12D knockdown. (C) FCM analysis showing that cell cycle progression in ZC3H12D‐knockdown MDA‐MB‐468 cells. (D) Expression of cell cycle‐promoting mRNAs was measured by qRT‐PCR in MDA‐MB‐468 cells after ZC3H12D knockdown. (E) Half‐lives of CCND1 mRNA were measured in ZC3H12D silenced MDA‐MB‐468 cells. (F) mRNA expression of CCND1 gene was measured by qRT‐PCR after infecting huCCND1‐targeting shRNA/lentivirus in MDA‐MB‐468/shZC3H12D cells. (G) FCM analysis showing that cell cycle progression in MDA‐MB‐468 cells after co‐knockdown of ZC3H12D and CCND1. (H) Tumor growth curve was recorded (*n* = 6/group). (*p < 0.05 in unpaired *t*‐test.) (I) Immunoblotting analysis of ZC3H12D expression in MDA‐MB‐468 tumor tissues. (J) MDA‐MB‐468 tumors were collected and pictured after the mice sacrificed (*n* = 6/group). (K) H&E staining of lung tissue sections from nude mice bearing MDA‐MB‐468 tumors. Scale bar, 200 μm. Data are presented as mean ± SD; *p < 0.05, **p < 0.001, ***p < 0.0001 in unpaired *t*‐test.

We further verified the role of ZC3H12D in regulating breast tumor cell cycle progression by knocking down ZC3H12D in MDA‐MB‐468 and MCF7 cells via infection with an shRNA‐expressing lentivirus. ZC3H12D protein expression was reduced by approximately 69% and 77% by the two shRNAs, respectively (Figure 5A). As expected, ZC3H12D knockdown significantly promoted breast tumor cell proliferation (Figures 5B and S4A) and cell cycle progression (Figures 5C and Figure S4B), as shown by cell counting and flow cytometry assays. Although ZC3H12D expression was already lower in breast tumor cells, further knockdown of ZC3H12D significantly increased the mRNA expression of cell cycle‐promoting genes, including CCND1 (Figures 5D and S4C). Interestingly, the expression of cell cycle‐promoting genes was also upregulated in ZC3H12A−/− CD4^+^T cells and MEFs (mouse embryonic fibroblasts) (Figure S4D,E) [28, 29]. In addition, the half‐lives of cell cycle‐promoting mRNAs were prolonged in breast tumor cells after ZC3H12D was knocked down (Figures 5E and S4F). To further confirm the involvement of cell cycle‐promoting genes in ZC3H12D‐induced cell cycle arrest in breast tumors, the CCND1 gene was effectively silenced in ZC3H12D‐knockdown MDA‐MB‐468 cells (Figure 5F). As a consequence, the percentage of cells in G1 phase returned to a level comparable to that of the control group after the double knockdown of CCND1 and ZC3H12D (Figure 5G). These results confirmed that ZC3H12D induces breast tumor cell cycle arrest by inhibiting the expression of cell cycle‐promoting genes. We also confirmed that ZC3H12D knockdown promoted breast tumor growth (Figure 5H–J) and metastasis (Figure 5K) in vivo. These results indicated that reducing ZC3H12D expression indeed promoted cell cycle progression in breast tumor cells and in vivo tumor progression by increasing the stability of cell cycle‐promoting mRNAs.

### 3.6 The RNA Helicase DDX5 Binds to the Common Stem–Loop Structure to Counteract ZC3H12D‐Mediated Decay of Cell Cycle‐Promoting mRNAs

**FIGURE 6:**
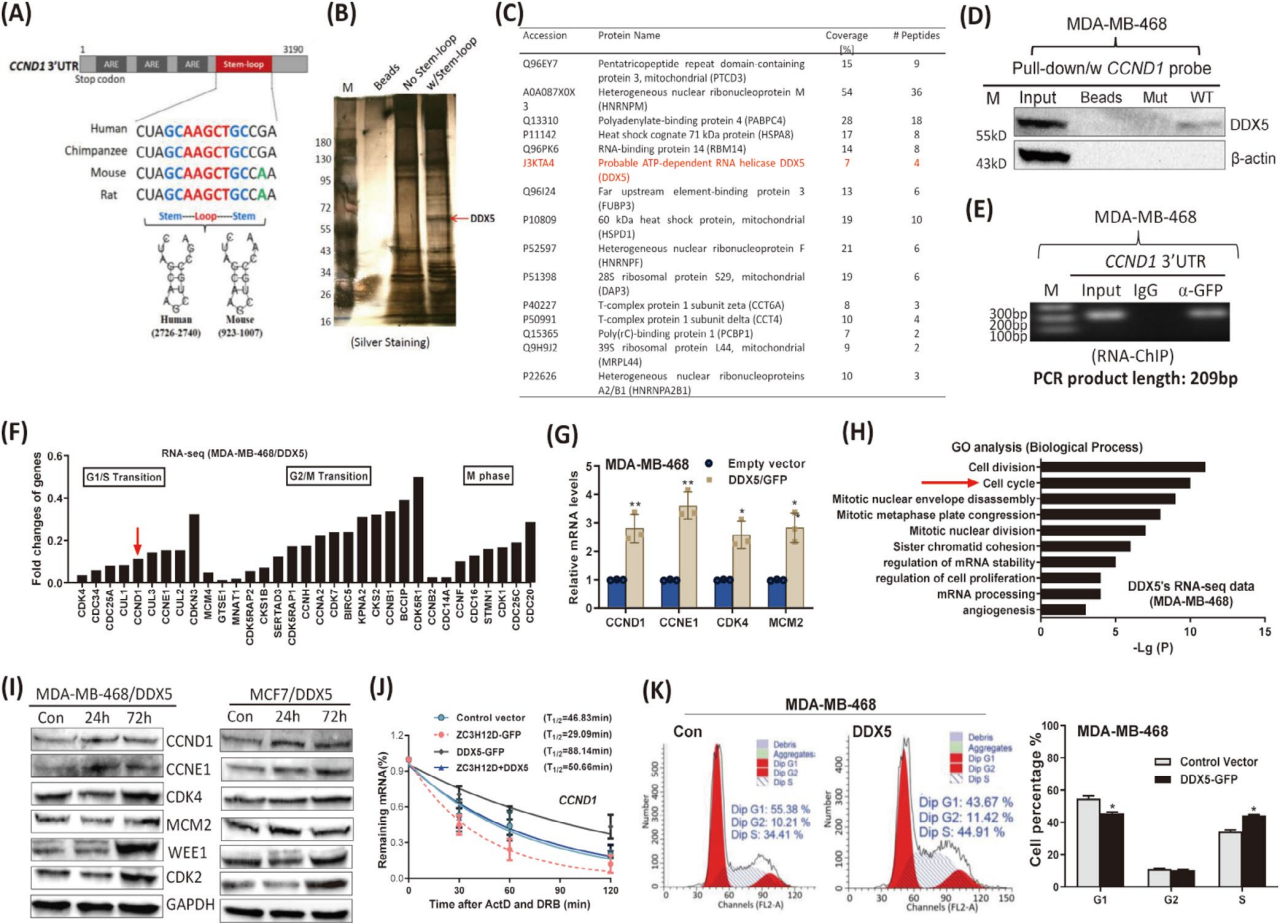
The RNA helicase DDX5 binds to the common stem–loop structure to counteract ZC3H12D‐mediated decay of cell cycle‐promoting mRNAs. (A) Alignment of CCND1 3′UTRs among different species and schematic representation of stem–loop structures. (B) Silver staining of gel separating immunoprecipitated proteins by CCND1 stem–loop RNA probes (w/stem–loop) in MDA‐MB‐468 cells. (C) Proteins most likely to interact with *CCND1* stem–loop structure identified by mass spectrometry. (D) RNA pull‐down followed by immunoblotting showing the interaction between DDX5 and *CCND1* stem–loop structure in MDA‐MB‐468 cells. (E) RIP‐ChIP analysis indicating the endogenous interaction between DDX5 and *CCND1* stem–loop structure in MDA‐MB‐468/DDX5‐GFP cells. (F) RNA sequencing data showed that the cell cycle‐promoting mRNAs were upregulated by DDX5 in MDA‐MB‐468 cells. (G) mRNA expression of cell cycle‐promoting genes was measured by qRT‐PCR in DDX5‐overexpressing MDA‐MB‐468 cells. (H) GO analysis of the regulated genes by DDX5 in MDA‐MB‐468 cells. The ‘cell cycle’ term was indicated by red arrow. (I) Expression of cell cycle‐promoting proteins in DDX5‐overexpressing MDA‐MB‐468 and MCF7 cells as measured by immunoblotting. (J) Half‐lives analysis of *CCND1* mRNA after ZC3H12D and DDX5 overexpression in MDA‐MB‐468 cells. (K) FCM analysis showing that cell cycle progression in DDX5‐overexpressing MDA‐MB‐468 cells. Data are represented as mean ± SD. *p < 0.05, **p < 0.01 in unpaired *t*‐test.

The 3′UTR is known to determine the turnover of mRNAs [30]. Several types of *Cis*‐acting elements are present in the 3′UTR, such as AU‐rich element (ARE) [31], GU‐rich element (GRE) [32], and stem–loop structure [33], which can be bound by RBPs to stabilize or degrade mRNAs. Interestingly, a conserved 3′UTR sequence among different species was identified to be folded into a stem–loop structure, and even a base was changed in mouse and rat sequences (Figure 6A), indicating that this conserved stem–loop structure might be important for controlling CCND1 expression. We previously confirmed that ZC3H12D bound to the stem–loop structure to induce the decay of the human CCND1 mRNA. Given the high expression of the CCND1 mRNA in breast tumor cells, a consideration of other proteins that bind to this stem–loop structure for mRNA stabilization is reasonable. To identify these potential interacting proteins, RNA pull‐down and mass spectrometry experiments were performed using biotin‐labeled stem–loop probes and linearized RNA probes (heated to 95°C and then rapidly cooled to delete the RNA secondary structure). As shown in Figure 6B, several clear and unique protein bands were enriched by the RNA probes with stem–loop structures compared with the group enriched with probes without stem–loop structures. Notably, many RBPs, such as DDX5, poly(A)‐binding protein 4 (PABPC4), and RNA‐binding motif protein 14 (RBM14), were identified and listed according to their molecular weights and number of peptides, further confirming our RNA pull‐down results (Figure 6C and Table S5). DDX5 was chosen for a subsequent study to validate its interaction with the CCND1 stem–loop structure. Indeed, DDX5 was detected in the proteins pulled down by probes containing stem–loop structures but not in the linearized probe group, as shown by RNA pull‐down followed by Western blotting (Figure 6D). Beta‐actin was used as a negative control. Next, an RNA immunoprecipitation–chromatin immunoprecipitation (RIP‐CHIP) assay was performed and revealed that the CCND1 stem–loop sequence could be amplified from the precipitated RNAs with an anti‐GFP antibody but not an isotype control IgG antibody (Figure 6E), indicating that DDX5 bound to the stem–loop structure of CCND1 inside cells. We further examined the effect of DDX5 on CCND1 mRNA expression in breast tumor cells. DDX5 upregulated the expression of a series of cell cycle‐promoting genes, including CCND1 (Figures 6F,G and S5A). The results of the GO analysis also revealed that DDX5 regulated the expression of cell cycle‐related genes in breast tumor cells (Figure 6H). Notably, most of the cell cycle‐promoting genes upregulated by DDX5, including *CCND1*, *CDK4*, *CUL3* (*cullin‐3*), *CCNE1*, were downregulated by ZC3H12D (Figure 2D), indicating that ZC3H12D and DDX5 jointly regulate the expression of cell cycle‐promoting genes in opposite ways. Moreover, the protein expression of CCND1 and other cell cycle‐promoting genes was also increased by DDX5 in breast tumor cells (Figure 6I). To define the effects of DDX5 on ZC3H12D‐induced CCND1 mRNA degradation, we overexpressed DDX5 in ZC3H12D‐overexpressing breast tumor cells and then performed an mRNA stability assay. Overexpression of DDX5 can attenuate ZC3H12D‐induced suppression of CCND1 mRNA in breast tumor cells (Figure S5B). Compared with that in control cells, the half‐life of the CCND1 mRNA in DDX5‐overexpressing breast tumor cells was prolonged. Importantly, DDX5 overexpression could increase the half‐lives of *CCND1*, *CCNE1*, and *CDK4* mRNAs, and counteract the shortened half‐life of the CCND1 mRNA caused by ZC3H12D (Figures S5C and 6J). As expected, DDX5 promoted the G1/S cell cycle transition of breast tumor cells (Figure 6K). Overall, we discovered that the RNA helicase DDX5 could bind to the stem–loop structure in the 3′UTR of the *CCND1* mRNA to increase its stability, allowing it to resist the ZC3H12D‐induced mRNA degradation in breast tumor cells.

### 3.7 DDX5 and ZC3H12D Antagonistically Regulate CCND1 Expression in Human Breast Tumors

**FIGURE 7:**
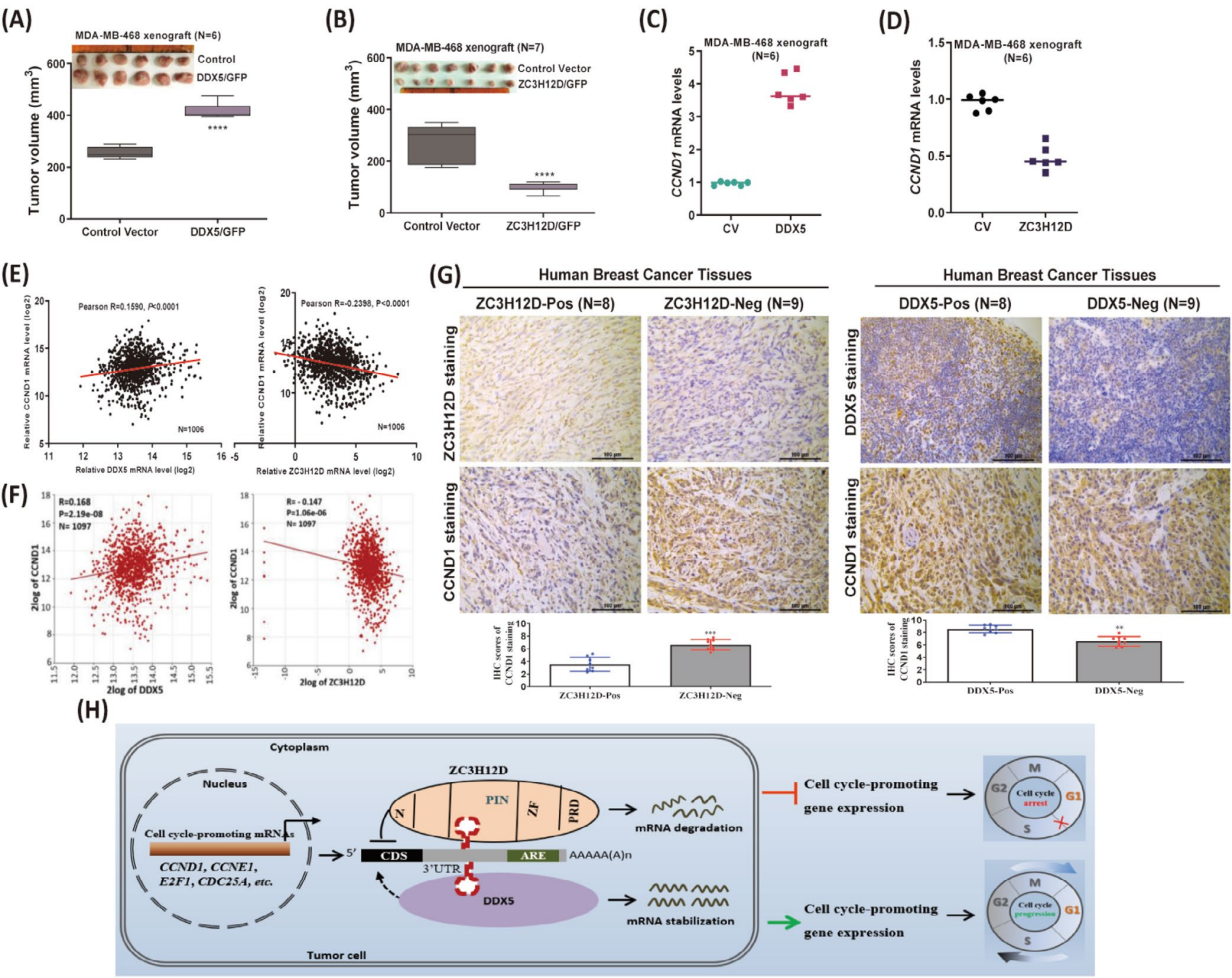
DDX5 and ZC3H12D antagonistically regulate CCND1 expression in human breast tumors. (A, B) Tumor volume of DDX5‐overexpressing xenograft tumors (A) and ZC3H12D‐overexpressing xenograft tumors (B) was recorded after the mice sacrificed. (C, D) Expression of CCND1 mRNA was measured by qRT‐PCR in DDX5‐overexpressing (C) and ZC3H12D‐overexpressing (D) xenografts and their control groups, respectively. (E, F) Pearson's correlation analysis between *DDX5*, *ZC3H12D* and *CCND1* expression in human breast cancer patients were conducted based on OncoLnc (www.oncolnc.org) (E) and R2 (http://r2.amc.nl) (F) online tools. (G) Representative IHC images of breast tumor specimens. Scale bars, 50 or 100 μm. (H) Schematic model showing the regulation mechanism of ZC3H12D and DDX5 on cell cycle progression in breast tumor cells. Data are shown as mean ± SD; **p < 0.001, ***p < 0.0001, ****p < 0.00001 in unpaired *t*‐test.

Next, we explored the roles of DDX5 and ZC3H12D in breast tumor progression. MDA‐MB‐468 cells overexpressing DDX5 or ZC3H12D were inoculated into the mammary glands of nude mice, respectively. The sizes of tumors overexpressing ZC3H12D were significantly reduced, whereas DDX5 effectively increased the tumor sizes, compared with those of control tumors (Figure 7A,B). To determine whether DDX5 expression was correlated with human breast cancer aggressiveness, we analyzed different human breast cancer datasets. In contrast to the ZC3H12D expression pattern, DDX5 protein expression was significantly increased in human breast tumor tissues (Figure S6A,B), including luminal, HER2+, and TNBC subtypes (Figure S6C). Additionally, DDX5 protein expression was higher in all stages of breast cancer (Figure S6D). Moreover, increased *DDX5* expression was significantly correlated with a poor clinical prognosis (Figure S6E). We subsequently analyzed the effects of ZC3H12D and DDX5 on CCND1 mRNA expression in human breast tumors. Interestingly, CCND1 mRNA expression was significantly decreased in MDA‐MB‐468 tumors overexpressing ZC3H12D, while it was significantly increased in DDX5‐overexpressing xenografts (Figure 7C,D). Moreover, the expression of *CCND1* in human breast tumor tissues decreased with increasing ZC3H12D expression but increased with increasing DDX5 expression (Figure 7E,F). The histologic analysis further revealed that the degree of CCND1 staining was lower (CCND1‐negative) in breast tumor tissues with high ZC3H12D staining (ZC3H12D‐positive), but greater CCND1 staining (CCND1‐positive) was observed in breast tumor tissues with greater DDX5 staining (DDX5‐positive) (Figure 7G). In addition, DDX5 and ZC3H12D were also positively and negatively correlated with the expression of several cell cycle‐promoting genes, such as WEE1 and CDK2, in human breast tumor tissues (Figure S6F,G). Therefore, we proposed a model to elucidate the potential antagonistic roles of ZC3H12D and DDX5 in regulating the cell cycle progression of breast tumor cells (Figure 7H). ZC3H12D degraded the mRNAs of CCND1 and other cell cycle‐promoting genes by binding to the conserved stem–loop structure localized in the 3′UTR via its RNase domain, thereby inhibiting their expression and the G1/S phase transition of breast tumor cells. On the other hand, the RNA helicase DDX5 stabilized the CCND1 mRNA by binding to the common stem–loop structure and promoted its expression and tumor cell cycle progression. In conclusion, our findings established that ZC3H12D and DDX5 play distinct anticancer and carcinogenic roles by modulating cell cycle progression in breast tumors. In the future, inducing ZC3H12D expression while suppressing DDX5 expression in breast tumors may be a promising strategy for targeted cell cycle therapy.

## 4 Discussion

Many RBPs are abnormally expressed in human cancers and are involved in regulating cancer progression as oncogenes or tumor suppressors [34]. In this study, for the first time, we elucidated the expression patterns of RBPs in human breast cancer tissues. Our study revealed that the expression of the endonuclease ZC3H12D and the RNA helicase DDX5, two RBPs, was significantly repressed and increased in human breast cancer tissues, respectively. Both RBPs affected cell cycle progression in breast tumors. The difference is that ZC3H12D inhibits the G1/S cell transition in breast tumor cells, whereas DDX5 promotes this transition. Interestingly, cell cycle‐promoting genes are commonly targeted and regulated by ZC3H12D and DDX5 but in positive and negative manners, respectively. To our knowledge, although many RBPs have been shown to cooperatively regulate target gene expression, few studies have reported how these RBPs antagonistically regulate their expression, especially the expression of cell cycle‐related genes in human breast cancer [27, 35]. Therefore, our results revealed a unique function of RBPs in the regulation of cell cycle‐related gene expression.

CCND1 is an important cell cycle‐promoting gene, and its expression is tightly controlled at the posttranscriptional level [36]. Many RBPs are involved in regulating CCND1 gene expression by controlling mRNA stability [37, 38]. Here, we showed that ZC3H12D downregulated the expression of CCND1 by degrading its mRNA through its RNase domain. In fact, this result is not surprising, as previous studies demonstrated that ZC3H12 family proteins degrade the mRNAs of their target genes, such as IL6 [39], c‐Rel [29], and B‐cell lymphoma‐2 like 1 (BCL2L1) [17]. It has been reported that many miRNAs can also regulate the mRNA stability of *CCND1* by directly targeting the 3′ UTR, such as miRNA‐195 [40] and miRNA‐193b [41]. We speculated that miRNAs or other RBPs might collaborate with ZC3H12D/DDX5 to regulate the mRNA stability of CCND1, further investigation would be needed to confirm this.

Additionally, our findings here confirmed that the ribosomal protein RPL4 is critical for the ZC3H12D‐mediated decay of the CCND1 mRNA. The data provided in this work and other works [28] suggest that ribosomal proteins can facilitate the mRNA degradation by ZC3H12 family proteins. As a ubiquitously expressed protein, further research is still needed to elucidate whether there are tissue‐specific cofactors interacting with ZC3H12D in different cellular or tissue contexts. The identification of cell cycle‐promoting mRNAs that can be specifically degraded by ZC3H12D adds new substrates for ZC3H12 family proteins. We speculated that ZC3H12D selectively targets the cell cycle‐promoting transcripts, which may be a result of the long‐term evolution of life. The high expression of the CCND1 mRNA in breast tumor cells led us to look for potential RBPs that could compensate for the degradation by ZC3H12D. Based on this idea, we identified the RNA helicase DDX5 as a CCND1 mRNA stabilizer. Previous and recent studies have shown that DDX5 can maintain mRNA stability [42, 43], which further supports our conclusion. Overall, we provided evidence that the RBPs ZC3H12D and DDX5 antagonistically modulate CCND1 mRNA stability and expression at the posttranscriptional level. Notably, our RNA‐seq data provided evidence that ZC3H12D and DDX5 may not only antagonize the mRNA stability and expression of the CCND1 gene but also regulate the expression of additional cell cycle‐promoting genes. Thus, more studies are needed to further explore the roles and mechanisms of ZC3H12 family proteins and DDX proteins in different contexts.

The RNA structure is an important *cis*‐acting element that is recognized and bound by RBPs to control gene expression [44]. Here, we showed that a conserved RNA stem–loop structure is present in the 3′UTRs of CCND1 across different species. Our results demonstrate that this stem–loop structure is indispensable for ZC3H12D‐induced CCND1 mRNA degradation, which is consistent with previous reports describing an interaction between ZC3H12 family proteins and stem–loop structures [17]. Interestingly, the results of this study showed that DDX5 can also bind to this common structural RNA element in the CCND1 3′UTR. Notably, DDX5 is believed to be a splicing regulator and interacts with the RNA stem–loop structure at the 5′ splice site of *tau* exon 10 [45]. Recent reports have shown that R‐loops are recognized and bound mainly by DDX5, indicating that DDX5 is likely to target structural RNA [46, 47]. These reports further support our findings. DDX5 expression is abnormally high in human breast cancers and can promote tumor progression [48, 49]. Interestingly, DDX5 has been reported to regulate tumor cell cycle progression [50], which is consistent with our results. Overall, we unveiled a novel regulatory network in which ZC3H12D and DDX5 antagonistically regulate the mRNA stability and expression of cell cycle‐promoting genes in breast tumor cells. Our findings also provide potential targets and strategies for breast cancer treatment in the future.

## 5 Conclusions

Overall, our results demonstrate that RBPs ZC3H12D and DDX5 antagonize breast tumor cell cycle progression by competitively regulating the mRNA stability and expression of cell cycle‐promoting genes via targeting the RNA stem–loop structure, which broadens our understanding of the RBP‐mediated post‐transcriptional regulation of cell cycle‐related genes. These findings reveal a novel reciprocal modulation of tumor cell cycle transition and highlight the potential of ZC3H12D and DDX5 as a pair of promising therapeutic targets for breast cancer.

## Author Contributions

**Liang Sun:** data curation (lead), formal analysis (lead), investigation (equal), methodology (equal), validation (equal), visualization (equal), writing – original draft (equal). **Xueting Liu:** methodology (equal). **Wenbao Lu:** conceptualization (lead), funding acquisition (lead), project administration (lead), supervision (lead), writing – review and editing (lead).

## Ethics Statement

This study was conducted in accordance with the Declaration of Helsinki and approved by the Experimental Animal Care and Ethics Committee of the Institute of Microcirculation, Chinese Academy of Medical Sciences and Peking Union Medical College.

## Conflicts of Interest

The authors declare no conflicts of interest.

## Supporting information


**Data S1:** cam471396‐sup‐0001‐Supinfo1.docx.

**Figure S1:** ZC3H12D is downregulated in human breast tumor tissues and is positively related to patient survival.
**Figure S2:** ZC3H12D regulates the expression of cell cycle‐related genes and inhibits cell proliferation in breast tumor cells.
**Figure S3:** ZC3H12D specifically degrades cell cycle‐promoting mRNAs in a stem–loop structure‐dependent manner to induce cell cycle arrest via the RNase domain.
**Figure S4:** Knocking down ZC3H12D increases the stability and expression of cell cycle‐promoting mRNAs.
**Figure S5:** The RNA helicase DDX5 counteracts ZC3H12D‐mediated inhibition of cell cycle‐promoting mRNAs.
**Figure S6:** DDX5 and ZC3H12D antagonistically regulate CCND1 expression in human breast tumors.
**Table S1:** RNA‐seq analysis of human breast tumor cells overexpressing ZC3H12D.
**Table S2:** Identify of genes interacting with ZC3H12D in breast tumor cells by irCLIP.
**Table S3:** Identify of proteins interacting with ZC3H12D in breast tumor cells by Mass Spectrometry.
**Table S4:** Identify of proteins interacting with CCND1 stem‐loop structure in breast tumor cells by Mass Spectrometry.
**Table S5:** RNA‐seq analysis of human breast tumor cells overexpressing DDX5.
**Table S6:** PCR Primer and RNA‐EMSA Probes Sequences.

## Data Availability

All data supporting the findings of this study are included in the article and/or the Supporting Information. The RNA sequencing data from this study are deposited in the Sequence Read Archive (SRA) database under projects PRJNA1178540, PRJNA1178802 and PRJNA1179509 (CLIP‐seq). The MS data are available via ProteomeXchange with the IDs: PXD057000.
