# Supplementary material for: ZC3H12D and DDX5 Antagonistically Regulate Cyclin D1 mRNA Stability and Cell Cycle Progression in Breast Cancer
Source: Cancer Med. 2025 Nov 20;14(22):e71396. doi: 10.1002/cam4.71396 (PMC12631744; doi:10.1002/cam4.71396)
Supplement: Supplementary file 1 — Data S1: cam471396‐sup‐0001‐Supinfo1.docx. [file CAM4-14-e71396-s001.docx]

**Supplementary Material S1**

**Bioinformatic analysis of RBPs expression in human breast tumor datasets**

Human breast tumor datasets were downloaded from the TCGA database (https:// tcga-data.nci.nih.gov/tcga/). The expression matrix was selected only from the primary tumors and their adjacent health tissues. The known 1756 genes coding for RNA-binding proteins were analyzed. The limma package of R studio (version 4.0.0) was used for differential analysis of target genes (cutoff：|log2FC|>1 & FDR<0.05).

**Antibodies and reagents**

Monoclonal mouse anti-E2F1 (66515-1-Ig), polyclonal rabbit anti-ZC3H12D (24991-1-AP), anti-p21 (10355-1-AP), anti-DDX5 (10804-1-AP), anti-CCND1 (26939-1-AP), anti-CCNE1 (11554-1-AP), anti-CDK4 (11026-1-AP), anti-MCM2 (10513-1-AP), anti-WEE1 (29474-1-AP), anti-CDK2 (10122-1-AP), and anti-RPL4 (11302-1-AP) antibodies were from Proteintech (Cook County, IL, USA). Mouse anti-CDKN3 (sc-135864) antibody was from Santa Cruz. Polyclonal rabbit anti-PARP1 (9542S), anti-Caspase3 (9662S) antibodies were from Cell Signaling Technology (Danvers, MA, USA). Polyclonal rabbit Anti-GFP (A-11122) were from ThermoFisher (Waltham, MA, USA). Isotype normal rabbit IgG (ab37415) was from Abcam (Cambridge, UK). GAPDH (AC002) and β-actin (AC004) antibodies were from ABclonal Company (Wuhan, China). G418 (G8168), puromycin (P8833), actinomycin D (ActD, A1410), and 5, 6-Dichlorobenzimidazole 1-β-D-ribofuranoside (DRB, D1916) were from Sigma-Aldrich (St. Louis, USA). Protein A/G PLUS-Agarose beads were from Sant Cruz Biotechnology (Dallas, Texas, USA). GFP-coated beads (gta-20) were from ChromoTek (Munich, Germany). Dynabeads M-280 streptavidin (11205D) were from Invitrogen (Grand Island, USA).

**RNA isolation and qRT-PCR**

Total RNA of breast tumor tissues or cultured cells were extracted with TRIzol reagent (Invitrogen, Grand Island, USA) and reverse transcribed to cDNA. qPCR was performed using SYBR green Fast Master Mix (#04913850001; Roche, Basel, Switzerland). The relative gene expression of interest genes was analyzed based on the 2-∆∆Ct method and normalized to GAPDH.

**Western Blotting**

Cell or tissue samples were collected and lysed with a modified RIPA buffer containing PMSF (#10837091001) and protease inhibitor cocktail (#04693159001; Roche, Basel, Switzerland). Equal amount of protein lysates was loaded and resolved by SDS-PAGE. The protein band was detected with HRP-conjugated secondary antibodies using ECL chemiluminescent detection method (#34077; Pierce, Rockford, IL, USA).

**shRNA lentivirus and adenovirus**

Two lentiviral shRNAs (TRCN0000239339; TRCN0000239342) targeting human ZC3H12D mRNA, two lentiviral shRNAs (TRCN0000117637; TRCN0000429089) targeting human RPL4 mRNA, and one lentiviral shRNA targeting human CCND1 (TRCN0000295873) were purchased from Merck (Kennyworth, NJ, USA). A scramble control shRNA was used as a control. Lentiviral particles were packaged in HEK293T cells by cotransfecting shRNA-pLKO.1, pCMV-dR8.2, and pMD2.G constructs. After two rounds infection, the target cells were selected with puromycin (1.0 µg/mL) for two weeks. ZC3H12D/GFP-expressing adenovirus, DDX5/GFP-expressing adenovirus or GFP-expressing control adenovirus were packaged by GeneChem Company (Shanghai, China).

**RNA immunoprecipitation (RIP)**

Zc3h12d/GFP fusion protein-expressing MDA-MB-468 cells were lysed and total protein extracts were precleared with isotype IgG, and then incubated with anti-GFP antibody or isotype IgG for 4 hours at 4℃. The protein/RNA complexes were immunoprecipitated with protein A/G agarose beads. The bound RNA was extracted with TRIzol from the pulled down RNA-protein complexes and reverse transcribed to cDNA for RT-PCR detection.

**Luciferase reporter assay**

The luciferase reporters containing full-length or mutants of *CCND1*, *CCNE1*, *CDK2*, or *MCM2* 3'UTRs were transfected into HEK293 cells with ZC3H12D-GFP and GFP expression constructs, respectively. The luciferase activity was measured according to the methods of Dual-Luciferase Reporter Assay System (Promega, Madison, WI, USA). All transfections were repeated at least three times.

**Immunocytochemistry**

MDA-MB-231 cells were fixed in 4% paraformaldehyde in PBS for 15 min, and then permeabilized in 0.5% Triton X-100 in PBS for 10 min. Cells were washed three times and blocked in 1.0% BSA for 30 min at room temperature. Anti-ZC3H12D and anti-RPL4 primary antibodies were used for staining in combination with secondary antibodies conjugated to FITC or Alexa Fluor 555 (Invitrogen, Grand Island, USA). 4',6-Diamidino-2-phenylindole (DAPI) was used for nucleus staining. Images were captured on a Zeiss microscope.

**RNA immunoprecipitation-chromatin immunoprecipitation (RIP-ChIP)**

DDX5/GFP fusion protein-expressing DMA-MB-468 cells were cultured and cross-linked for 10 min by addition of formaldehyde (1% v/v), and then crosslinking were stopped by adding glycine (125 mM; Sigma-Aldrich, Saint Louis, MO, USA). Cells were washed with cold PBS and resuspended in 500 μL of polysome lysis buffer. The cell lysates were collected, sonicated, and pre-clear with rabbit IgG to remove non-specific background. Pre-cleared lysates were used for IP with anti-GFP antibody coated beads or isotype IgG-coated beads at 4℃ for 4 h. After pull-down, 100 μL supernatants were taken out for Input. RNA was isolated with Trizol reagent, and re-suspended in 50 μL of RNase-free water, followed by RT-PCR detection.

**Tumor tissue samples**

A total of seventeen human breast tumor samples and their matched surrounding ‘healthy’ tissues were obtained previously. The tumors have mixture molecular subtypes, including eight luminal A, three luminal B, and four triple negative breast tumors. The subtypes of the rest three tumors are unknown. Among these tumors, eight samples were evaluated as higher ZC3H12D expression (ZC3H12D-positive) and nine samples showed lower ZC3H12D expression (ZC3H12D-negative) according to IHC scoring. Eight samples were evaluated as higher DDX5 expression (DDX5-positive) and nine samples showed lower DDX5 expression (DDX5-negative). All subjects gave their informed consent for inclusion before they participated in the study. The study was conducted in accordance with the Declaration of Helsinki, and the experimental protocols were approved by the Medical Ethics Committee of the Institute of Microcirculation, CAMS & PUMC.

***CCND1* 3'UTR stem‒loop structure prediction**

For 3'UTR stem‒loop structure sequence conservation analysis of *CCND1*, the 3'UTR sequences were extracted for different species from the National Center for Biotechnology Information (NCBI) database: *CCND1* 3'UTR: human (*Homo sapiens*; accession number NM_053056.3), chimpanzee (*Pan troglodytes*; XM_016921434.1), mouse (*Mus musculus*; NM_007631.2), rat (*Rattus*; NM_171992.4). Stem‒loop sequence conservation analysis was performed using DNAMAN software. The stem‒loop structure was predicted through RNAfold web server ( <http://rna.tbi.univie.ac.at/> ).
