# Supplementary material for: ZC3H12D and DDX5 Antagonistically Regulate Cyclin D1 mRNA Stability and Cell Cycle Progression in Breast Cancer
Source: Cancer Med. 2025 Nov 20;14(22):e71396. doi: 10.1002/cam4.71396 (PMC12631744; doi:10.1002/cam4.71396)
Supplement: Supplementary file 2 — Figure S1: ZC3H12D is downregulated in human breast tumor tissues and is positively related to patient survival. Figure S2: ZC3H12D regulates the expression of cell cycle‐related genes and inhibits cell proliferation in breast tumor cells. Figure S3: ZC3H12D specifically degrades cell cycle‐promoting mRNAs in a stem–loop structure‐dependent manner to induce cell cycle arrest via the RNase domain. Figure S4: Knocking down ZC3H12D increases the stability and expression of cell cycle‐promoting mRNAs. Figure S5: The RNA helicase DDX5 counteracts ZC3H12D‐mediated inhibition of cell cycle‐promoting mRNAs. Figure S6: DDX5 and ZC3H12D antagonistically regulate CCND1 expression in human breast tumors. Table S1: RNA‐seq analysis of human breast tumor cells overexpressing ZC3H12D. Table S2: Identify of genes interacting with ZC3H12D in breast tumor cells by irCLIP. Table S3: Identify of proteins interacting with ZC3H12D in breast tumor cells by Mass Spectrometry. Table S4: Identify of proteins interacting with CCND1 stem‐loop structure in breast tumor cells by Mass Spectrometry. Table S5: RNA‐seq analysis of human breast tumor cells overexpressing DDX5. Table S6: PCR Primer and RNA‐EMSA Probes Sequences. [file CAM4-14-e71396-s002.docx]

**Fig. S1.**


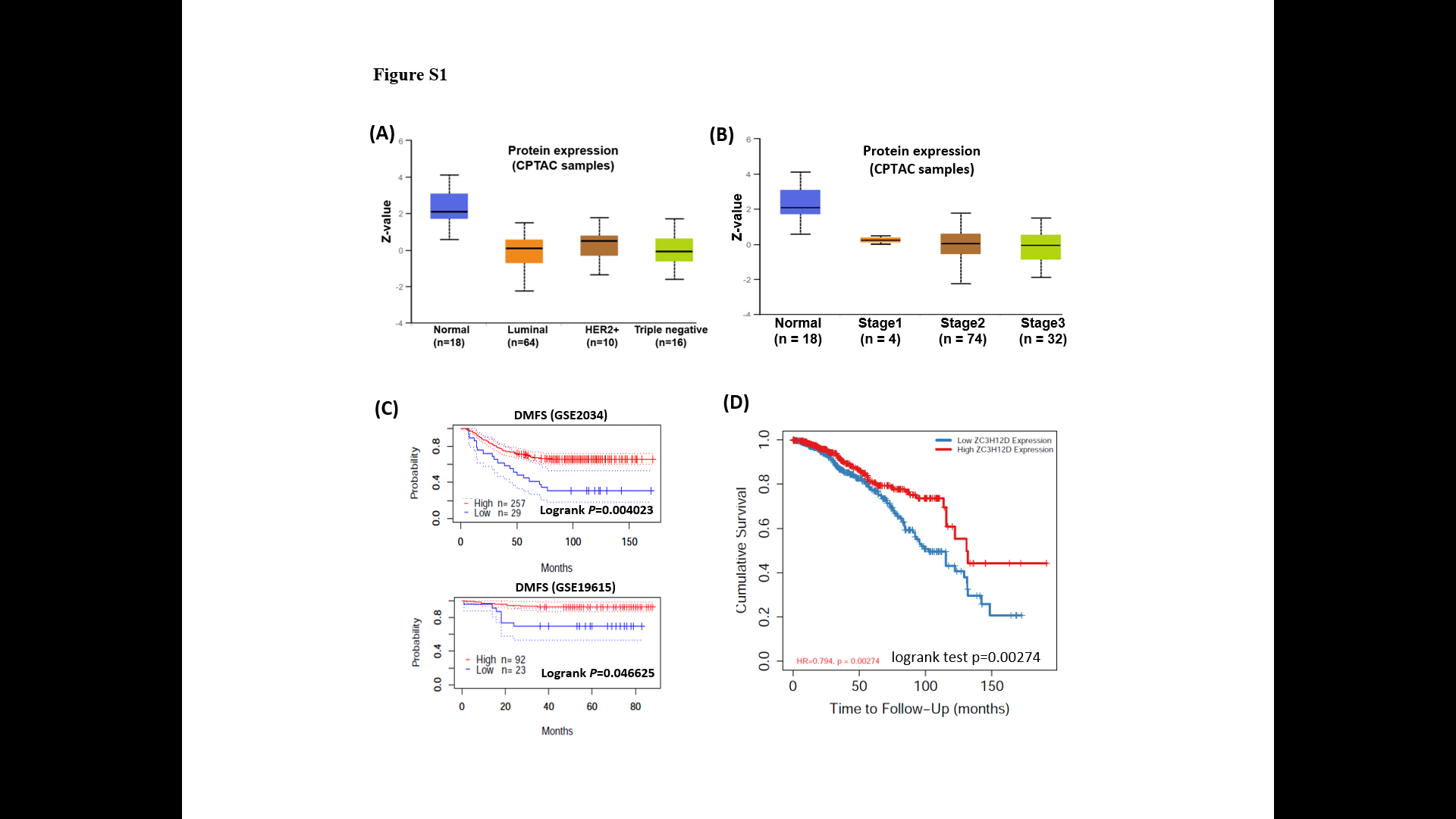


**Fig. S1. ZC3H12D is downregulated in human breast tumor tissues and is positively related to patient survival**

**A** Protein expression of ZC3H12D in normal breast tissues and different subtypes of human breast tumors tissues was displayed by UALCAN (<http://ualcan.path.uab.edu/analysis-prot.html>). **B** ZC3H12D protein expression in diffident stages of human breast tumors and normal tissues was displayed using the UALCAN online tool. **C** DMFS (Distant Metastasis Free Survival) curves for ZC3H12D-low and ZC3H12D-high breast cancer patients (PrognoScan). **D** Relapse-Free Survival curves of breast cancer patients with low and high ZC3H12D transcripts (TIMER2.0).

**Fig. S2.**


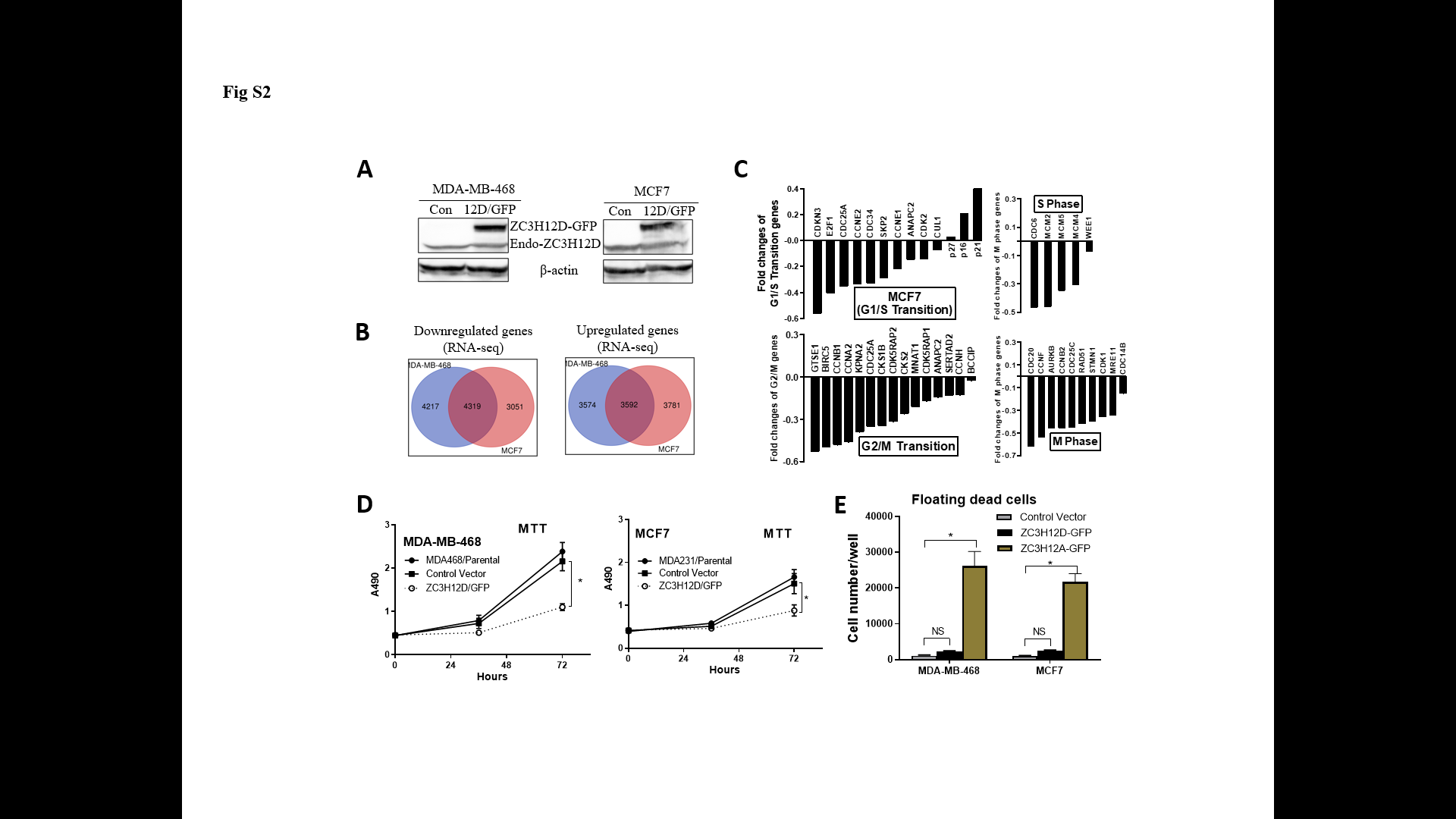


**Fig. S2. ZC3H12D regulates the expression of cell cycle-related genes and inhibits cell proliferation in breast tumor cells**

**A** Immunoblotting analysis of ZC3H12D-GFP fusion protein expression with anti-ZC3H12D antibody in MDA-MB-468 and MCF7 cells. **B** Venn diagrams showing the commonly down-regulated genes and up-regulated genes by ZC3H12D in human breast tumor cells. **C** Expression of cell cycle-related genes in ZC3H12D-overexpressing MCF7 cells as measured by RNA-seq. **D** MTT assay showing that cell activity of MDA-MB-468 and MCF7 cells after ZC3H12D overexpression. **E** Floating dead cells were counted in cultured MDA-MB-468 cells and MCF7 cells with ZC3H12D or ZC3H12A overexpression. Data are shown as mean ± SD; * *P* < 0.05 in unpaired *t*-test.

**Fig. S3.**


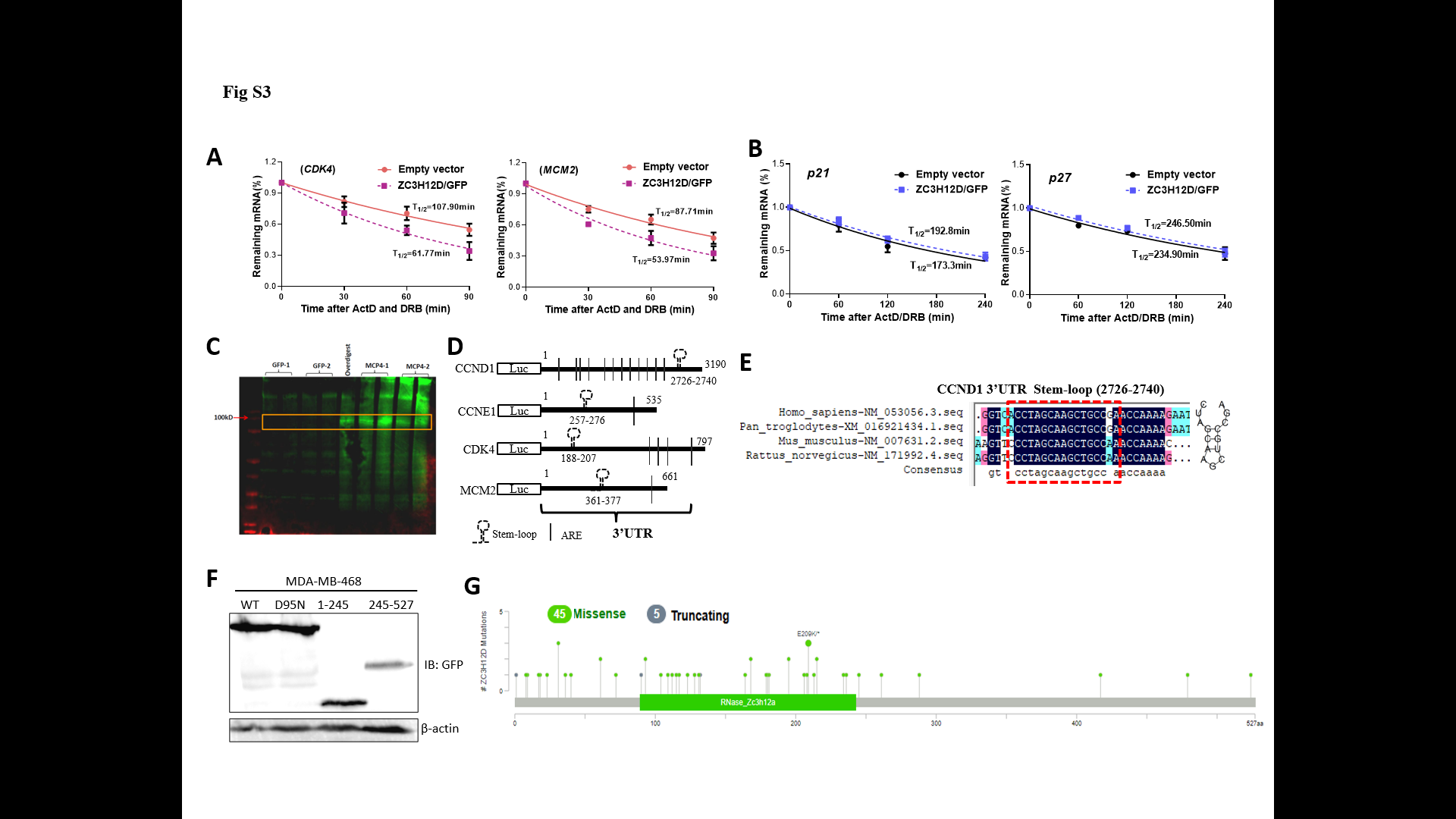


**Fig. S3. ZC3H12D specifically degrades cell cycle-promoting mRNAs in a stem‒loop structure-dependent manner to induce cell cycle arrest via the RNase domain**

**A, B** Half-lives of CDK4, MCM2 mRNAs (**A**) and p21, p27 mRNAs (**B**) were measured in MDA-MB-468 cells after ZC3H12D overexpression. C SDS-PAGE imaging of RNA bound by ZC3H12D-GFP (MCPIP4-GFP) in irCLIP assay. **D** Schematic representation of the luciferase reporters containing 3'UTRs of cell cycle-related mRNAs. **E** The 3'UTR sequences from different species for CCND1 gene were aligned using DNAMAN software. The stem‒loop sequences were predicted by RNAfold web server and indicated by red box. **F** Immunoblotting analysis showing the protein expression of ZC3H12D truncations. **G** The mutation sites of ZC3H12D in human breast cancer were displayed using cBioPortal.

**Fig. S4.**


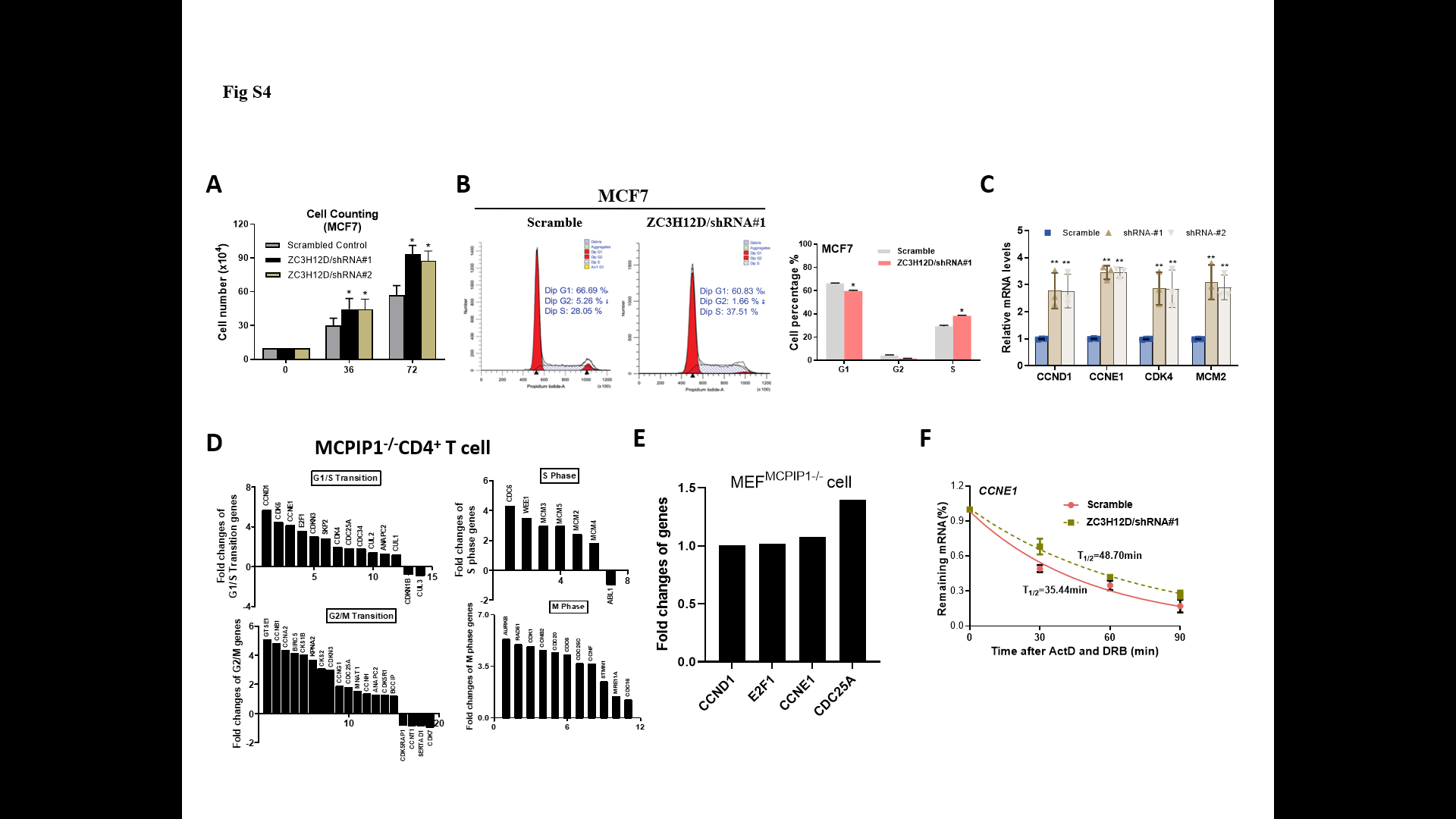


**Fig. S4. Knocking down ZC3H12D increases the stability and expression of cell cycle-promoting mRNAs**

**A** Cell counting showing that cell proliferation of MCF7 cells after ZC3H12D knockdown. **B** FCM analysis showing that cell cycle progression in ZC3H12D knocked down MCF7 cells. Percentages of cells in the G1, G2, and S phase were analyzed. **C** Expression of cell cycle-promoting mRNAs was measured by qRT-PCR in MCF7 cells after ZC3H12D knockdown. **D, E** Expression of the indicated cell cycle-related mRNAs was analyzed in MCPIP1-/- CD4+T cells (**D**) and MEFs (**E**), respectively. **F** Half-lives of *CCNE1* mRNA were measured in MDA-MB-468 cells after ZC3H12D knockdown. Data are shown as mean ± SD; * *P* < 0.05, ** *P* < 0.001 in unpaired *t*-test.

**Fig. S5.**


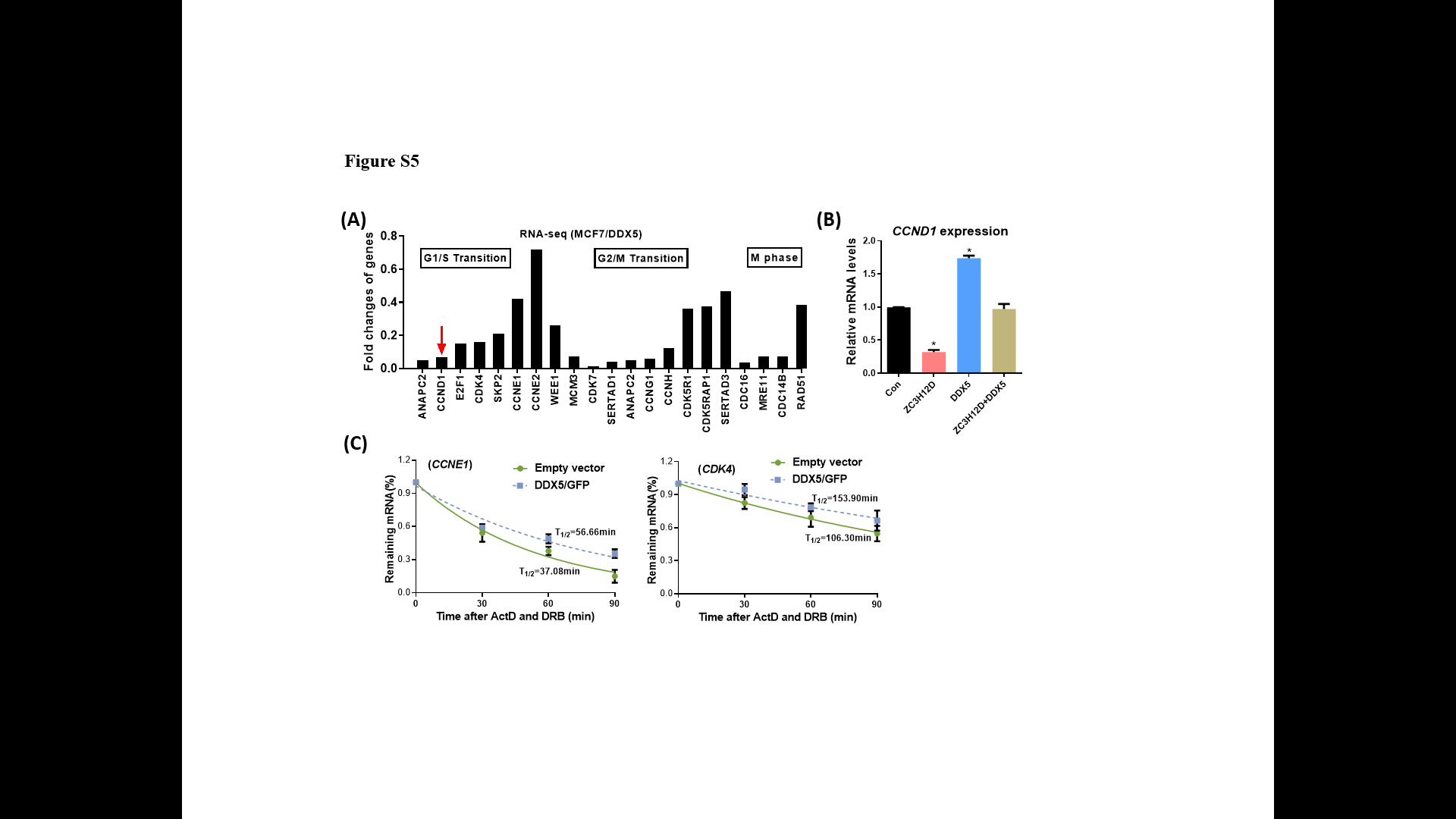


**Fig. S5. The RNA helicase DDX5 counteracts ZC3H12D-mediated inhibition of cell cycle-promoting mRNAs**

**A** Expression of cell cycle-related genes in DDX5-overexpressing MCF7 cells as measured by RNA-seq. **B** qRT-PCR analysis showing mRNA expression of *CCND1* in ZC3H12D-overexpressing, DDX5-overexpressing, and ZC3H12D/DDX5 co-overexpressing MDA-MB-468 cells, respectively. **C** Half-lives of *CCNE1* and CDK4 mRNAs were measured in DDX5-overexpressing MDA-MB-468 cells. Data are shown as mean ± SD; * *P* < 0.05 in unpaired *t*-test.

**Fig. S6.**


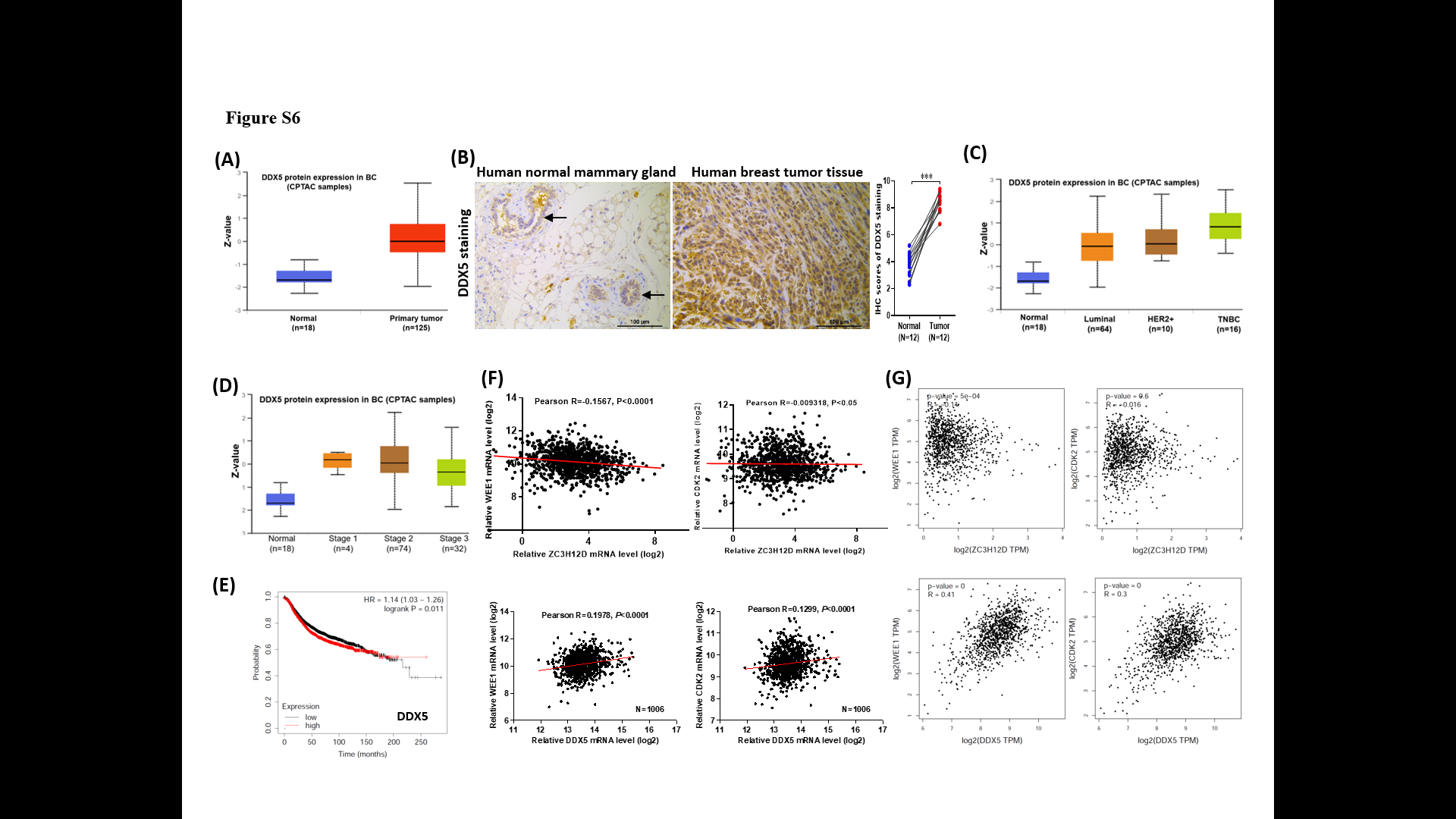


**Fig. S6 DDX5 and ZC3H12D antagonistically regulate CCND1 expression in human breast tumors**

**A** Protein expression of *DDX5* in human primary breast tumors and normal tissues (<http://ualcan.path.uab.edu/analysis-prot.html>). **B** Representative immunohistochemistry (IHC) images showing DDX5 protein expression in normal mammary gland tissues and breast tumor tissues (left). Scale bar, 100μm. IHC analysis of DDX5 staining in 12 pairs of matched breast tumor and normal tissues (right). **C** Protein expression of *DDX5* in different subtypes of breast cancer (<http://ualcan.path.uab.edu/analysis-prot.html>). **D** Protein expression of *DDX5* were analyzed according to the main pathological stages of breast cancer (<http://ualcan.path.uab.edu/analysis-prot.html>). **E** Relapse-free survival curve of breast cancer patients with low and high tumor DDX5 transcripts. **F, G** Pearson's correlation analysis between *ZC3H12D*, *DDX5* and *WEE1*, *CDK2* expression in human breast cancer patients were performed with OncoLnc (<http://www.oncolnc.org/>) (**F**) or R2 (<http://r2.amc.nl>) (**G**) online tools. Data are shown as mean ± SD; *** *P* < 0.0001 in unpaired *t*-test.
